# Reprogramming innate immune memory in bladder cancer: the proposed bone marrow–bladder axis and next-generation BCG-based immunotherapies

**DOI:** 10.3389/fimmu.2026.1945559

**Published:** 2026-10-06

**Authors:** Meng Yang, Yu Guo, Yu-zhao Shen, Yan-Li Sun, Dai-Ju Ma, Yuan-Jia Tang

**Affiliations:** 1 Department of Geriatric, Xiangyang No. 1 People’s Hospital, Hubei University of Medicine, Xiangyang, Hubei, China; 2 Department of Colorectal and Anal Surgery, Xiangyang No. 1 People’s Hospital, Hubei University of Medicine, Xiangyang, Hubei, China; 3 Department of Urology, Xiangyang No. 1 People’s Hospital, Hubei University of Medicine, Xiangyang, Hubei, China; 4 Department of General Practice, Xiangyang No. 1 People’s Hospital, Hubei University of Medicine, Xiangyang, Hubei, China; 5 Department of Emergency, Xiangyang No. 1 People’s Hospital, Hubei University of Medicine, Xiangyang, Hubei, China

**Keywords:** BCG immunotherapy, BCG resistance, bladder cancer, precision immunotherapy, trained immunity

## Abstract

Intravesical Bacillus Calmette–Guérin (BCG) remains the standard adjuvant treatment for high-risk non-muscle-invasive bladder cancer (NMIBC), yet the mechanisms underlying its durable clinical benefit and treatment resistance remain incompletely understood. Although BCG has traditionally been regarded as a local immunotherapy, emerging clinical and preclinical evidence suggests that intravesical BCG can induce trained-immunity-associated systemic immune changes, including alterations in circulating innate immune responses and hematopoietic programming. In this Review, we propose the bone marrow–bladder axis as a hypothesis-generating conceptual framework linking local microbial sensing with systemic hematopoietic responses that may contribute to sustained myeloid and adaptive antitumor immunity. Building upon this framework, we further introduce the Failure-to-Train (FTT) model, which proposes that selected immune-training-related mechanisms may contribute to heterogeneous patterns of BCG failure through disruption of productive immune training at distinct stages of the proposed immune-training circuit. Finally, we discuss the implications of this framework for biomarker development, experimental validation and mechanism-guided immunotherapy. By considering systemic immune training as a potential contributor to BCG responses, this Review provides a conceptual framework for investigating treatment response and resistance and for developing testable strategies for next-generation immunotherapy.

## 1 Introduction

Among solid tumors, bladder cancer has long served as a paradigm for successful cancer immunotherapy. Following complete transurethral resection, intravesical Bacillus Calmette–Guérin (BCG) remains the standard bladder-preserving treatment for patients with high-risk and selected intermediate-risk non-muscle-invasive bladder cancer (NMIBC) (1–3). Unlike conventional cytotoxic therapies, BCG mediates its antitumor effects primarily through activation of host immunity within the bladder, making NMIBC one of the earliest and most successful clinical models of cancer immunotherapy (1, 2). Despite its long-standing clinical success, therapeutic responses remain highly heterogeneous. While many patients achieve durable disease control, a substantial proportion experience recurrence or progression, fail to respond despite adequate BCG therapy, or discontinue treatment because of toxicity (2, 4). These clinical observations highlight a fundamental unresolved question: why does the same intravesical microbial therapy induce long-lasting antitumor immunity in some patients but only transient or ineffective immune responses in others? Addressing this question is essential for understanding BCG responsiveness, overcoming treatment resistance, and guiding the development of next-generation immunotherapies.

The conventional paradigm attributes BCG efficacy primarily to local immune activation within the bladder, involving microbial sensing, inflammatory mediator release, innate immune-cell recruitment, antigen presentation, and subsequent adaptive antitumor responses (1, 5, 6). However, this local inflammatory model does not fully explain several key clinical observations. Durable immune protection frequently persists far beyond the lifespan of mature innate immune cells. Furthermore, repeated maintenance instillations are required to achieve optimal therapeutic benefit, and systemic immune alterations have been detected following an ostensibly local treatment (2, 4, 7–9). Moreover, the intensity of acute inflammation correlates poorly with long-term clinical outcome, suggesting that transient immune activation alone is insufficient to account for the durability and heterogeneity of BCG responses. These observations indicate that a broader conceptual framework is needed to explain how transient local microbial stimulation is converted into durable systemic antitumor immunity.

The emerging concept of trained immunity provides a compelling framework to address these unresolved questions. Unlike classical adaptive immune memory, trained immunity is characterized by long-lasting functional reprogramming of innate immune cells and hematopoietic stem and progenitor cells (HSPCs) through coordinated epigenetic, transcriptional, and metabolic remodeling (10–13). BCG is among the best-characterized inducers of trained immunity and has been shown to induce persistent innate immune reprogramming beyond its immediate antimicrobial effects (10, 13). Importantly, prospective studies in patients with NMIBC have shown enhanced heterologous cytokine responsiveness of peripheral blood immune cells for several months after intravesical BCG, providing evidence of persistent systemic innate immune alterations after treatment (7, 8). However, these observations do not by themselves establish that such systemic changes are required for clinical antitumor efficacy. More recently, Daman and colleagues provided evidence linking intravesical BCG to systemic hematopoietic reprogramming in experimental models, with supportive observations in patient samples (9). Together, these findings support the possibility that intravesical BCG influences systemic innate immunity and hematopoiesis in addition to its established local effects, although the contribution of these processes to clinical efficacy in NMIBC remains to be determined.

Based on these observations, we propose the bone marrow–bladder axis as a hypothesis-generating conceptual model that integrates local bladder immunity with systemic hematopoietic responses. Within this model, inflammatory and microbial signals generated after intravesical BCG are hypothesized to influence bone marrow hematopoietic stem and progenitor cells (HSPCs) and trained myelopoiesis, while bone marrow-derived myeloid cells may subsequently contribute to local immune activation, antigen presentation, and adaptive antitumor immunity (1, 9, 10, 13, 14). Importantly, the individual components of this proposed circuit are supported by different levels of evidence, and the complete bidirectional sequence has not yet been prospectively demonstrated as an integrated mechanism in patients with NMIBC. Throughout this Review, we therefore distinguish direct observations in patients with NMIBC from mechanistic evidence obtained in bladder-cancer experimental models, supporting evidence from systemic BCG vaccination or other trained-immunity settings, and conceptual inferences derived from integration of these evidence streams. Within this framework, we further propose the Failure-to-Train (FTT) model as a conceptual approach for organizing immune-training-related mechanisms that may contribute to BCG resistance, including defects in microbial engagement, hematopoietic reprogramming, myeloid differentiation and trafficking, and innate-to-adaptive immune communication. The FTT framework is intended to generate testable mechanistic hypotheses rather than to represent an established clinical classification or a comprehensive explanation for all forms of BCG treatment failure.

In this Review, we integrate emerging evidence on trained immunity with recent advances in bladder cancer immunology to develop a conceptual framework for BCG immunotherapy. We first distinguish transient innate activation from durable trained-immunity-associated responses and examine the bladder as a potential mucosal environment for local immune programming. We then discuss evidence that intravesical BCG may influence systemic hematopoietic responses and consider how these changes could contribute to sustained myeloid function and adaptive antitumor immunity. Building on this framework, we introduce the FTT model to organize selected immune-training-related mechanisms of BCG resistance and heterogeneity, and evaluate how recombinant BCG, trained-immunity agonists, cytokine-based therapies, immune-checkpoint blockade, metabolic interventions, biomaterial platforms, and biomarker-guided strategies might be investigated within this framework. Taken together, the proposed bone marrow–bladder axis and FTT model provide a hypothesis-generating framework for understanding BCG responsiveness and resistance and for guiding future mechanistic, biomarker, and therapeutic studies in bladder cancer (**Figure 1**).

**Figure 1:**
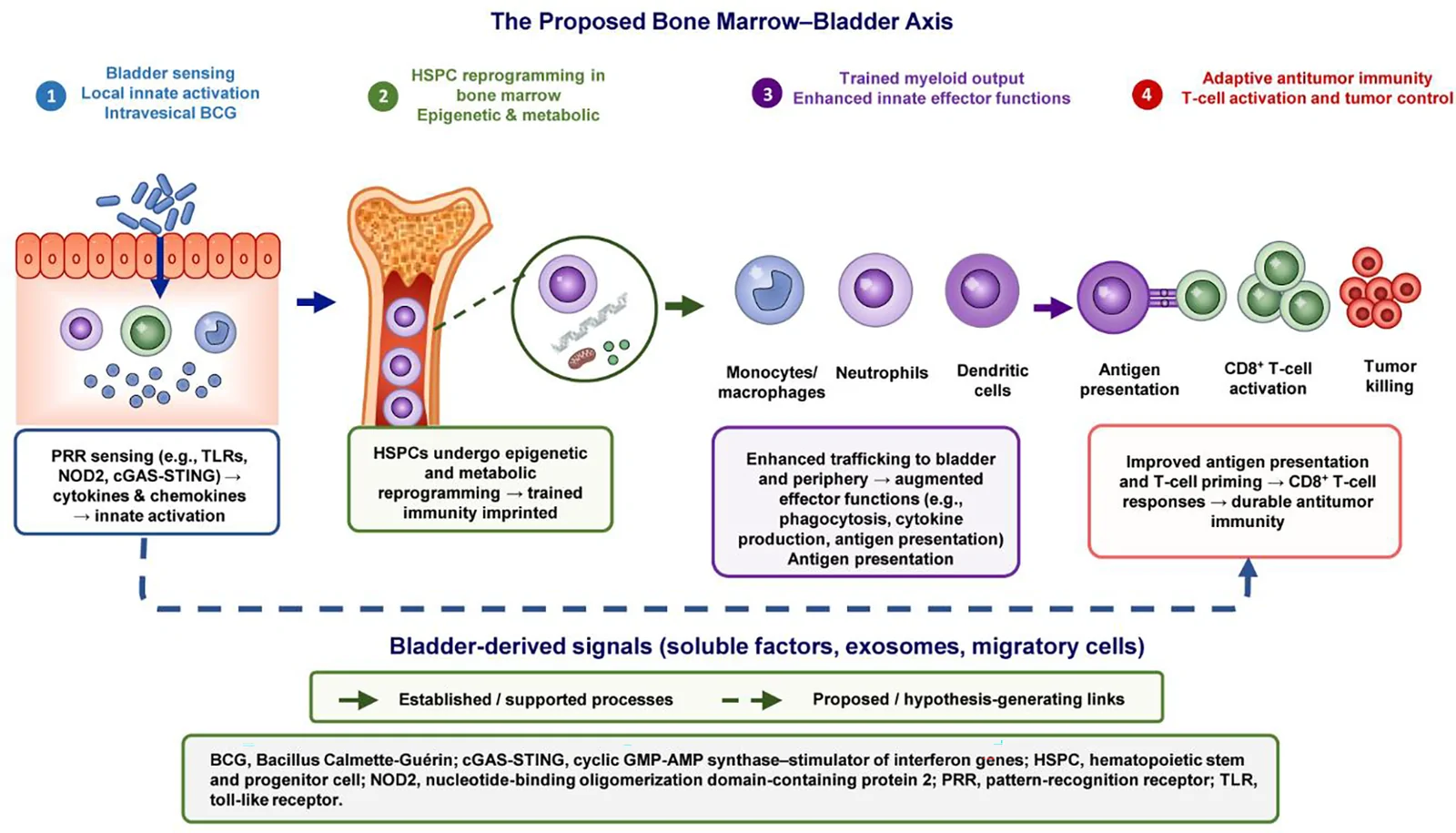
The proposed bone marrow–bladder axis: a conceptual framework of BCG-induced trained immunity in bladder cancer. Intravesical BCG initiates local innate immune activation through pattern-recognition pathways, inducing cytokine production and immune-cell recruitment within the bladder. Bladder-derived inflammatory, microbial and metabolic signals may communicate with the bone marrow, where hematopoietic stem and progenitor cells (HSPCs) undergo epigenetic, metabolic and transcriptional reprogramming, resulting in trained myelopoiesis. Trained-immunity-associated myeloid cells may subsequently contribute to bladder immune remodeling, antigen presentation and adaptive antitumor immunity. Together, these processes represent a proposed bone marrow–bladder immune-training circuit that may contribute to durable tumor control after BCG therapy. Disruption of this circuit may occur at multiple stages, including immune engagement, initiation, central training, myeloid execution and adaptive immune transfer, as conceptualized in the Failure-to-Train (FTT) framework. These processes represent hypothesis-generating mechanisms rather than validated biomarkers or clinical subtypes of BCG resistance.

## 2 Literature search and evidence selection

This review was conducted as a structured narrative review rather than a systematic review. Relevant literature was identified through searches of PubMed/MEDLINE, Web of Science, and Scopus from database inception to August 15, 2026. Search terms were used individually and in combination and included “bladder cancer, “ “non-muscle-invasive bladder cancer, “ “NMIBC, “ “Bacillus Calmette-Guérin, “ “BCG, “ “BCG resistance, “ “BCG-unresponsive, “ “trained immunity, “ “innate immune memory, “ “hematopoietic stem and progenitor cells, “ “HSPC, “ “myelopoiesis, “ “epigenetic reprogramming, “ “metabolic reprogramming, “ “tumor microenvironment, “ and “immune checkpoint.” Reference lists of relevant articles were also screened to identify additional studies.

The inclusion criteria comprised English-language original research articles, clinical trials, translational studies, and mechanistic studies relevant to BCG-induced immunity, trained immunity, bladder cancer immunobiology, treatment resistance, and emerging BCG-based therapeutic strategies. Animal and *in vitro* studies were included when they provided mechanistic evidence relevant to the proposed bone marrow–bladder axis or Failure-to-Train (FTT) framework. Clinical studies were prioritized when evaluating therapeutic efficacy, safety, biomarkers, and treatment outcomes. Narrative reviews were used primarily to provide background context and to identify relevant primary literature. Studies unrelated to the central themes of this review, non-English-language publications, conference abstracts, and preprints were excluded.

Studies were selected based on relevance to the major themes of the review, with consideration of mechanistic rigor, clinical relevance, methodological quality, and consistency with independent evidence. Particular attention was given to studies directly examining BCG-induced trained immunity, systemic innate immune reprogramming, HSPC responses, myeloid-cell remodeling, and innate-to-adaptive immune interactions. Because the purpose of this review was to integrate established and emerging evidence and develop a mechanistic conceptual framework rather than to perform quantitative evidence synthesis, no formal meta-analysis or systematic risk-of-bias assessment was undertaken. A summary of the literature-search and evidence-selection strategy is provided in **Supplementary Table S2**.

## 3 BCG immunotherapy in bladder cancer: from clinical success to mechanistic gaps

### 3.1 Clinical efficacy and limitations of BCG

Intravesical BCG remains the cornerstone of bladder-preserving therapy for patients with high-risk non-muscle-invasive bladder cancer (NMIBC) following complete transurethral resection of bladder tumor (15, 16). Current clinical guidelines recommend a six-week induction course followed by maintenance therapy for patients with carcinoma *in situ*, high-grade T1 disease, and selected recurrent or multifocal high-grade Ta tumors (15, 16). Although the optimal duration and intensity of maintenance remain subjects of ongoing debate, randomized clinical trials and meta-analyses consistently demonstrate that maintenance BCG provides substantially greater clinical benefit than induction therapy alone. In the landmark Southwest Oncology Group (SWOG) trial, maintenance BCG significantly prolonged recurrence-free survival, findings that were subsequently confirmed by conventional and individual patient-level meta-analyses showing reduced recurrence and disease progression compared with induction alone or intravesical mitomycin C (17–19). Collectively, these data indicate that repeated BCG exposure provides greater clinical benefit than induction alone and raise the possibility that sustained immunological remodeling contributes to long-term therapeutic efficacy.

Despite its long-standing clinical success, BCG therapy remains limited by substantial interpatient heterogeneity in treatment response. A clinically meaningful proportion of patients develop persistent high-grade disease, experience early recurrence after an initial response, or eventually progress to muscle-invasive bladder cancer despite receiving adequate BCG therapy (15, 16, 20). These patients are generally classified as having BCG-unresponsive disease, a category encompassing BCG-refractory tumors and selected early high-grade recurrences that are unlikely to benefit from additional BCG instillation (16, 20). For surgically eligible patients, radical cystectomy remains the preferred oncological treatment because it provides the greatest chance of durable disease control, although its considerable perioperative morbidity and profound impact on urinary, sexual, and overall quality of life limit acceptance by many patients (16, 20). Consequently, bladder-preserving alternatives-including immune-checkpoint inhibitors, intravesical gene therapy, IL-15-based immunotherapy, oncolytic viruses, and novel drug-delivery platforms-have expanded rapidly. Nevertheless, durable clinical benefit is still achieved in only a subset of patients, highlighting the need for a deeper mechanistic understanding of BCG responsiveness and resistance (20, 21).

The delivery of BCG therapy is further challenged by treatment-related toxicity, incomplete adherence, and fluctuations in global BCG supply. Most patients experience local cystitis-like symptoms, including urinary frequency, urgency, dysuria, hematuria, and pelvic discomfort, whereas systemic flu-like symptoms are less common. Serious complications, such as disseminated BCG infection, sepsis, pneumonitis, hepatitis, osteomyelitis, and vascular infection, are rare but may necessitate treatment discontinuation (2, 15). Consequently, completion of prolonged maintenance schedules is often difficult, particularly in older patients with comorbidities or limited physiological reserve. Consistent with this, only a minority of patients in the landmark SWOG maintenance trial completed the full three-year treatment schedule (17). In parallel, recurrent global BCG shortages have prompted dose reduction, abbreviated maintenance, and substitution with intravesical chemotherapy in many healthcare systems. Importantly, these practical constraints complicate the interpretation of treatment failure, as inadequate BCG exposure may mimic biological resistance, whereas continued instillation in truly resistant disease may unnecessarily delay more effective therapeutic intervention.

### 3.2 Limits of the conventional local-immune model

The conventional model of BCG immunotherapy centers on local immune activation within the bladder, whereby BCG–urothelial interactions trigger innate sensing, inflammatory mediator release, immune-cell recruitment, antigen presentation, and subsequent Th1-polarized antitumor responses (1, 5, 14). Although this paradigm explains the acute inflammatory and adaptive immune responses following BCG instillation, it does not fully account for the durability, schedule dependence, and systemic immune effects of BCG therapy.

Despite its broad acceptance, the conventional local-inflammatory paradigm leaves several fundamental mechanistic questions unresolved. Although BCG attachment, internalization, and pattern-recognition receptor signaling are considered essential initiating events, their relative contribution to clinical efficacy remains uncertain. Multiple innate sensing pathways—including TLR2, TLR4, TLR9, NOD2, inflammasome signaling, and the cGAS–STING axis—have all been implicated in BCG-induced immunity, yet no single pathway has emerged as a universal determinant of therapeutic response across different experimental systems, BCG strains, and patient populations (5, 14). Similarly, the identity of the dominant antitumor effector population remains incompletely defined. Although multiple innate and adaptive immune-cell populations contribute to BCG-induced immunity, their relative importance varies according to the tumor microenvironment, inflammatory context, and stage of treatment (1, 5, 14). These observations suggest that successful BCG immunotherapy is unlikely to depend on any individual receptor, cytokine, or immune-cell subset alone, but instead reflects coordinated interactions among multiple innate and adaptive immune compartments.

An even more important limitation of the conventional model is its inability to explain the durability and schedule dependence of BCG therapy. Acute inflammatory responses, including urinary cytokine release and cystitis-like symptoms, clearly indicate that the immune system has been activated, yet neither consistently predicts long-term clinical benefit. Conversely, robust acute inflammation does not necessarily translate into durable antitumor immunity. These observations suggest that the magnitude of inflammation alone is an inadequate surrogate for therapeutic efficacy and that the quality, persistence, and functional organization of the immune response are likely to be more important than its absolute intensity. This concept is further supported by the superior efficacy of maintenance BCG, which cannot be readily explained if each instillation merely represents an independent episode of transient inflammation. Instead, repeated instillations may progressively reinforce long-lasting immune remodeling, allowing immune responses to mature beyond acute activation while preserving durable antitumor function (17–19). Although direct mechanistic evidence remains incomplete, this interpretation provides a biologically plausible explanation for the schedule dependence of BCG therapy and naturally links repeated treatment to the emerging concept of trained immunity.

### 3.3 Mechanistic basis for a broader immune framework

Interpatient heterogeneity remains incompletely explained by conventional clinicopathological variables. Although tumor stage, grade, concomitant carcinoma *in situ*, tumor multiplicity, recurrence history, and adequacy of BCG exposure all influence outcome, they do not fully predict therapeutic response. Increasing evidence suggests that treatment efficacy is additionally shaped by tumor-intrinsic characteristics, host immune status, prior microbial exposure, urinary microbiota, metabolic conditions, and genetic variation in innate immune pathways (5, 20). Rather than acting through a single mechanism, these factors are likely to influence distinct stages of BCG immunotherapy, including microbial sensing, myeloid-cell recruitment, hematopoietic reprogramming, antigen presentation, and adaptive immune activation. Consequently, clinically similar cases of BCG failure may arise from distinct biological defects along the proposed immune-training axis.

Progress in defining these mechanisms has been further limited by the heterogeneous experimental models used to study BCG immunotherapy. Differences in BCG strains, treatment schedules, tumor models, and immune backgrounds complicate comparisons across studies. Moreover, subcutaneous and immune-deficient xenograft models fail to reproduce the bladder-specific immune microenvironment, whereas orthotopic models, although more physiologically relevant, remain technically variable. Most importantly, few studies simultaneously integrate tumor tissue, urine, peripheral blood, and bone marrow, making it difficult to reconstruct the complete trajectory of systemic immune training following intravesical BCG.

Collectively, these clinical and experimental limitations suggest that BCG efficacy cannot be fully explained by local nonspecific inflammation alone. A more comprehensive mechanistic framework should integrate treatment schedule, sustained immune remodeling, systemic hematopoietic responses, host heterogeneity, and coordinated innate–adaptive immune communication. In this context, the proposed bone marrow–bladder axis provides a unifying conceptual model linking local microbial sensing with systemic hematopoietic reprogramming, sustained trained myelopoiesis, and adaptive immune amplification. Importantly, this framework offers a plausible mechanistic context for investigating BCG responsiveness and selected immune-training-related mechanisms of treatment failure, thereby providing the conceptual foundation for the subsequent sections of this Review. Representative clinical and experimental evidence relevant to BCG-induced trained immunity and the proposed bone marrow–bladder axis is summarized in **Table 1**.

**Table 1:** Evidence supporting BCG-induced trained immunity and bone marrow–bladder axis.

Study	Study type/model	BCG exposure	Main immune findings	Evidence category for trained immunity	Relevance to the proposed bone marrow–bladder axis	Key limitation/interpretation
Kaufmann et al., 2018 (13)	Mouse model and human BCG vaccination	Systemic BCG vaccination	BCG reprogrammed HSPCs, enhanced myelopoiesis, and generated macrophages with persistent functional and epigenetic changes.	Extrapolative mechanistic evidence from systemic BCG settings; direct evidence of central trained immunity outside bladder cancer	Established the biological basis for HSPC-mediated persistence of BCG-induced innate immune memory.	Not performed in NMIBC; relevance to intravesical BCG therapy remains inferential.
van Puffelen et al., 2023 (7)	Prospective cohort of 17 patients with NMIBC	Intravesical induction and maintenance BCG	PBMCs showed increased TNF and IL-1β production after heterologous restimulation at 6–12 weeks; inflammasome-related epigenetic and transcriptional changes were detected.	Direct human evidence of peripheral trained immunity after intravesical BCG	Demonstrates that local bladder treatment can induce persistent systemic innate immune changes.	Limited cohort size; peripheral immune changes do not directly demonstrate bone marrow reprogramming.
Pichler et al., 2023 (8)	Longitudinal immune profiling in BCG-treated patients	Intravesical BCG	Altered chromatin accessibility and trained-immunity-associated innate responses were observed after treatment.	Supportive human evidence	Supports systemic immune reprogramming following local BCG treatment.	Specific contribution to antitumor efficacy and HSPC-level changes remain uncertain.
Atallah et al., 2024 (41)	Murine NMIBC models; trained macrophage and dendritic-cell experiments	Intravesical versus systemic BCG	Systemic BCG generated a more antitumoral immune environment; trained macrophages and dendritic cells enhanced tumor-specific CTL responses.	Mechanistic preclinical evidence linking innate training to adaptive immunity	Shows that the quality of systemic innate training may influence local tumor immunity.	Animal model evidence; translation to human NMIBC requires validation.
Daman et al., 2025 (9)	Orthotopic mouse models, HSPC transplantation, and patient samples	Intravesical BCG	BCG reached the bone marrow, reprogrammed HSPCs, amplified myelopoiesis, and generated functionally altered neutrophils, monocytes, and dendritic cells; trained HSPCs transferred enhanced tumor protection.	Mechanistic evidence from bladder cancer models with supporting human samples; strongest current evidence for central trained immunity in bladder cancer	Provides experimental support for the proposed bone marrow–bladder axis.	Integrated human validation of the complete circuit remains lacking.

Evidence levels are descriptive rather than formally graded. The table distinguishes direct human evidence, mechanistic evidence from bladder cancer models, extrapolative evidence from systemic BCG/trained-immunity settings, and conceptual implications for the proposed bone marrow–bladder axis.

## 4 From acute innate activation to trained immunity

### 4.1 Acute activation versus trained immunity

Intravesical BCG initially induces acute innate immune activation within the bladder, characterized by inflammatory mediator release and rapid immune-cell recruitment (1, 5).

Although this response is essential for initiating antitumor immunity, it should not be equated with trained immunity. Acute activation is transient and largely resolves after removal of the initiating stimulus, whereas trained immunity represents a persistent functional adaptation of innate immune cells and their progenitors that remains detectable after the initial inflammatory response has subsided (10, 11). Upon subsequent homologous or heterologous stimulation, trained cells exhibit altered responsiveness through durable transcriptional, epigenetic, and metabolic reprogramming rather than antigen-specific receptor rearrangement or clonal expansion (10, 22).

The distinction between acute activation and trained immunity is particularly relevant to BCG immunotherapy. Acute bladder inflammation reflects successful immune recognition but does not necessarily predict durable tumor control. One possibility is that durable clinical benefit is influenced by whether the initial inflammatory response is accompanied by sustained immune reprogramming. This conceptual transition—from transient activation to durable immune training—provides a plausible framework for examining whether repeated BCG exposure contributes to longer-lasting immune remodeling beyond acute inflammation. These distinct immune-response states are summarized in **Figure 2**.

**Figure 2:**
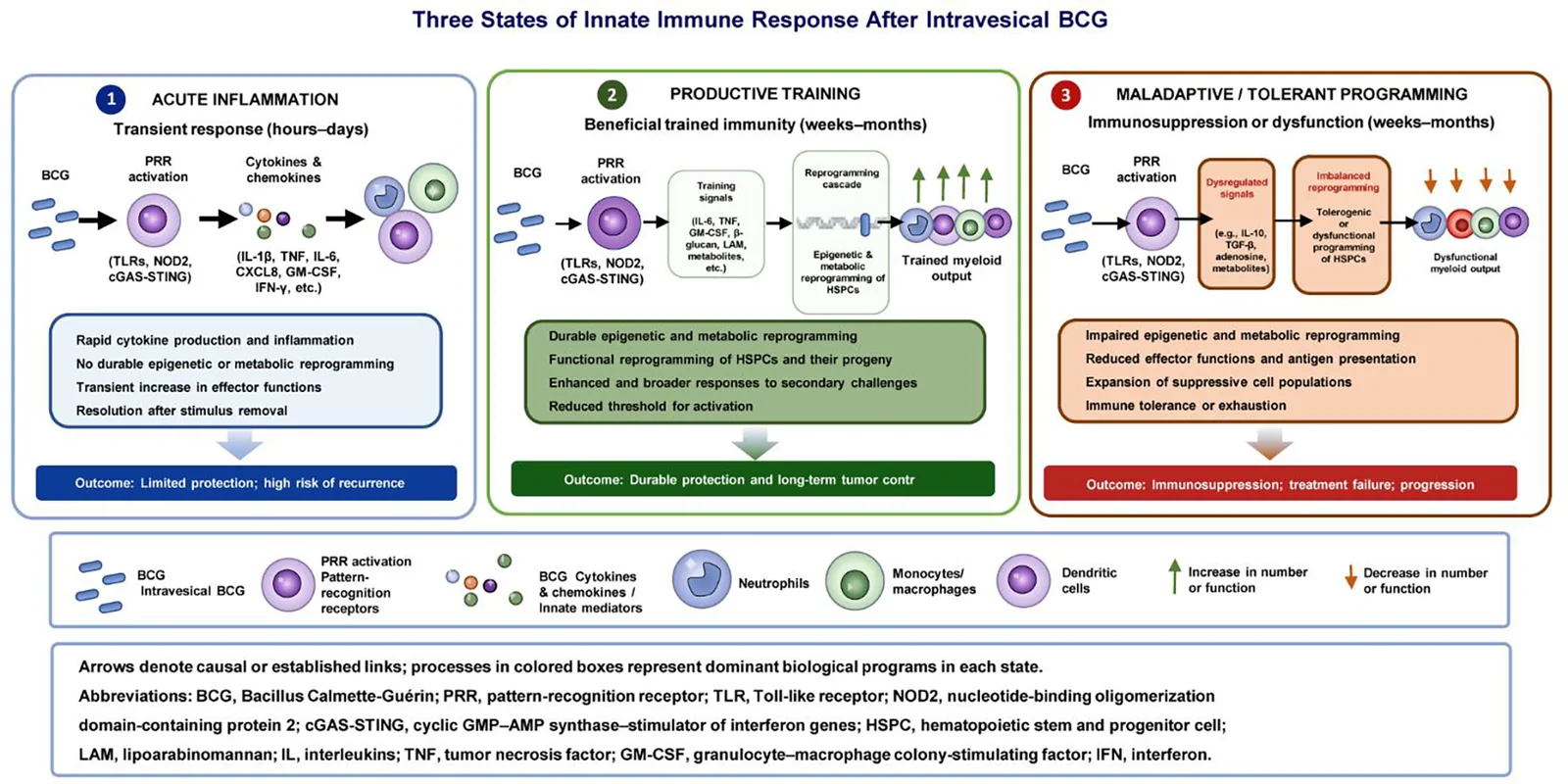
Acute inflammation versus trained immunity after intravesical BCG. Acute inflammation and trained immunity represent distinct biological outcomes following BCG stimulation. Acute inflammation is characterized by transient cytokine production, rapid recruitment of innate immune cells and short-lived effector responses that resolve after stimulus removal. In contrast, trained immunity involves durable epigenetic, metabolic and transcriptional reprogramming of immune cells, including hematopoietic progenitor compartments, resulting in enhanced responsiveness to secondary challenges. Depending on host and microenvironmental conditions, BCG stimulation may lead to transient activation without durable immune memory, productive trained-immunity-associated responses that may contribute to tumor control, or maladaptive immune states associated with dysfunctional or suppressive responses. These distinct states illustrate the context-dependent consequences of BCG-induced innate immune activation.

### 4.2 Epigenetic and metabolic reprogramming

Persistent innate immune memory is sustained by coordinated epigenetic and metabolic reprogramming rather than continuous inflammatory activation. Following exposure to BCG, β-glucan, and other microbial stimuli, innate immune cells acquire durable changes in chromatin accessibility and histone modifications at inflammatory and metabolic gene loci. Increased H3K4me3 at gene promoters and H3K27ac at active enhancers establish a transcriptionally permissive chromatin landscape, allowing trained cells to respond more rapidly and robustly upon secondary stimulation (22, 23). Importantly, these epigenetic alterations persist after the initial inflammatory response has resolved, distinguishing trained immunity from persistent inflammation. Rather than maintaining constitutive cytokine production, trained cells return to an apparently resting state while retaining latent regulatory programs that shape future responsiveness. Depending on the nature and duration of the primary stimulus, similar chromatin remodeling can also induce innate immune tolerance instead of enhanced responsiveness (10, 22–24).

Epigenetic remodeling is tightly coupled to cellular metabolism. Establishment of trained immunity requires metabolic rewiring toward increased glycolysis through the mTOR–HIF-1α pathway, while glutaminolysis, the tricarboxylic acid cycle, and the mevalonate pathway generate metabolites—including fumarate, succinate, acetyl-CoA, and α-ketoglutarate—that regulate chromatin-modifying enzymes and stabilize trained epigenetic states (25–27). Thus, metabolism provides not only the energy required for immune activation but also the substrates that directly control epigenetic programming. This interaction is particularly relevant in bladder cancer, where hypoxia, lactate accumulation, and altered lipid metabolism may reshape myeloid-cell function. Consequently, the outcome of BCG immunotherapy is likely determined not only by the intensity of initial receptor signaling but also by whether the local metabolic environment supports productive trained immunity or redirects innate immune cells toward immunosuppressive phenotypes.

### 4.3 Peripheral and central trained immunity

Although trained immunity was initially described in mature peripheral innate immune cells, accumulating evidence indicates that it operates at both peripheral and central levels. Peripheral trained immunity involves persistent functional reprogramming of circulating monocytes, macrophages, natural killer cells, and related innate immune populations. In humans, BCG vaccination induces long-lasting epigenetic remodeling of circulating monocytes, leading to enhanced cytokine production following homologous or heterologous stimulation and improved protection against unrelated infectious challenges, thereby providing the first clinical evidence that innate immune memory can persist beyond the initial inflammatory response (28). However, the relatively short lifespan of circulating monocytes and neutrophils indicates that durable trained immunity cannot be explained solely by changes in mature peripheral cells. This limitation has led to the concept of central trained immunity, whereby microbial stimulation reprograms hematopoietic stem and progenitor cells (HSPCs) within the bone marrow. Through transcriptional, epigenetic, and metabolic remodeling, trained HSPCs continuously generate functionally altered monocytes, neutrophils, dendritic cells, and other myeloid populations, thereby sustaining enhanced innate immune responsiveness long after the primary stimulus has resolved (13). Central training therefore provides a biological mechanism through which transient microbial exposure can produce durable systemic immune effects despite the rapid turnover of mature innate immune cells.

Evidence from patients with NMIBC indicates that intravesical BCG can induce immune responses extending beyond the bladder. Prospective studies demonstrated that peripheral blood mononuclear cells collected after intravesical BCG treatment exhibited enhanced TNF and IL-1β production following ex vivo stimulation with unrelated microbial ligands, with these functional changes persisting for several months after therapy (7). Although these studies demonstrated systemic innate immune adaptation, the cellular basis of this persistent response remained uncertain until recent work provided evidence that bone marrow HSPC reprogramming can contribute to trained myelopoiesis. More recently, Daman and colleagues provided direct mechanistic evidence in experimental models that intravesical BCG reprograms bone marrow HSPCs, promotes trained myelopoiesis, and generates functionally enhanced neutrophils, monocytes, and dendritic cells, with supportive observations in patient samples. Importantly, transplantation of HSPCs from BCG-treated mice transferred improved antitumor immunity to recipient animals, demonstrating that hematopoietic reprogramming is sufficient to sustain durable antitumor responses. These trained myeloid cells subsequently remodeled the tumor microenvironment, enhanced T-cell-dependent immunity, and potentiated immune-checkpoint blockade (9). These findings substantially expand the conventional view of BCG immunotherapy. Those support a model in which intravesical BCG may induce immune changes across the bladder, circulation, and bone marrow. In this model, central hematopoietic reprogramming provides a plausible mechanism that could contribute to the persistence and amplification of antitumor immunity.

### 4.4 Productive versus maladaptive immune training

The induction of innate immune memory is not invariably beneficial. Depending on the nature, intensity, and duration of the primary stimulus, together with the host metabolic state and tissue microenvironment, innate immune cells may undergo productive training, transient activation, or maladaptive reprogramming characterized by immune tolerance or immunosuppressive myelopoiesis (10, 24). Accordingly, trained immunity should be regarded as a dynamic spectrum of functional adaptation rather than a uniformly enhanced inflammatory response.

This distinction is particularly relevant to BCG immunotherapy. Acute bladder inflammation, urinary cytokine production, and leukocyte recruitment indicate successful immune activation but do not necessarily signify durable antitumor immunity. Productive trained immunity should generate functionally competent myeloid cells with enhanced tumor recognition, antigen presentation, and the capacity to support adaptive immune responses. Conversely, maladaptive training may sustain chronic inflammation without effective tumor control or promote immunosuppressive cell populations, including myeloid-derived suppressor cells and regulatory macrophages, thereby contributing to treatment failure (29).

These observations raise the hypothesis that one determinant of BCG outcome may be whether acute innate activation is accompanied by durable and appropriately regulated immune training. This conceptual distinction provides a rationale for the FTT framework proposed in this Review. The principal molecular mechanisms and biological consequences of BCG-induced trained immunity are summarized in **Table 2** and set the stage for the proposed bone marrow–bladder axis discussed in the following section.

**Table 2:** Molecular, metabolic, and cellular mechanisms contributing to BCG-associated trained immunity (10, 13, 22–27, 31).

Mechanistic level	Key mediators or processes	Functional consequence	Potential relevance to BCG therapy	Candidate readouts	Evidence status
Microbial sensing	TLR2, TLR4, TLR9, NOD2, cGAS–STING and inflammasomes	Initiation of inflammatory and antimicrobial signaling	May influence the strength and quality of the initial immune-training stimulus after BCG exposure	PRR expression; NF-κB activity; inflammasome activation	Human and preclinical evidence; direct contribution to trained immunity in NMIBC remains under investigation
Cytokine signaling	IL-1β, TNF, IL-6, GM-CSF, type I IFNs and IL-12	Myeloid activation, recruitment and differentiation	May connect local bladder sensing with peripheral and hematopoietic immune responses	Urinary/plasma cytokines; ex vivo restimulation assays	Human immune profiling and experimental evidence
Epigenetic remodeling	H3K4me3, H3K27ac and altered enhancer accessibility	Maintenance of inflammatory and metabolic loci in a poised state	May enable enhanced responses after secondary stimulation	ChIP-seq; CUT&Tag; ATAC-seq	Strong evidence from trained-immunity studies; NMIBC-specific validation remains limited
Glycolytic reprogramming	mTOR–HIF-1α signaling and increased glycolysis	Rapid ATP generation and biosynthetic support	May contribute to metabolic requirements associated with trained immune responses	ECAR; glucose uptake; lactate production	Extrapolative trained-immunity evidence with emerging cancer relevance
Mevalonate pathway	HMGCR and mevalonate-derived intermediates	Couples cholesterol metabolism to epigenetic and functional remodeling	May sustain innate immune training and membrane-dependent signaling	Metabolomics; HMGCR activity; sterol intermediates	Mechanistic evidence mainly from trained-immunity models; direct NMIBC evidence remains limited
Glutaminolysis and TCA remodeling	Glutamine utilization, fumarate, succinate and α-ketoglutarate	Regulation of histone-modifying enzymes and inflammatory gene accessibility	May link nutrient availability with persistent immune reprogramming	Isotope tracing; intracellular metabolite profiling	Experimental metabolic evidence; clinical relevance requires further validation
Transcriptional reprogramming	NF-κB, HIF-1α, C/EBPβ, PU.1, IRFs and STATs	Alteration of lineage commitment and effector programs	May shape trained myelopoiesis and antigen-presenting functions	RNA-seq; transcription-factor activity assays	Preclinical mechanistic evidence
HSPC reprogramming	Altered stem/progenitor states and lineage bias	Sustained generation of modified myeloid cells	May provide persistence beyond the lifespan of mature monocytes and neutrophils	Bone marrow single-cell profiling; circulating progenitor analysis	Preclinical bladder-cancer evidence; prospective human validation lacking
Trained myelopoiesis	Functionally modified monocytes, neutrophils and dendritic cells	Enhanced cytokine production, phagocytosis and antigen presentation	May contribute to recruitment and functional maintenance of antitumor myeloid populations in the bladder	Flow cytometry; killing assays; antigen-presentation assays	Preclinical evidence with emerging translational relevance
Innate-to-adaptive transfer	DC maturation, IL-12, CXCL9/CXCL10, MHC and costimulatory pathways	CD4+/CD8+ T-cell priming and memory formation	May facilitate transition from innate activation to adaptive antitumor responses	TCR sequencing; spatial immune profiling	Mechanistic evidence; complete bone marrow–bladder immune circuit remains unproven
Maladaptive programming	Immune tolerance, MDSCs, suppressive macrophages and tumor-promoting neutrophils	Chronic inflammation without effective tumor control	May contribute to toxicity, immune dysfunction and BCG resistance	Suppression assays; MDSC/Treg markers; basal versus restimulated cytokine responses	Conceptual framework supported by immunological evidence; requires prospective validation in BCG-treated NMIBC

Evidence categories reflect the current level of support and are not intended as formal evidence grading. Mechanistic links within the proposed bone marrow–bladder axis remain hypothesis-generating and require prospective validation.

## 5 The bladder as a mucosal training niche

### 5.1 Urothelial sensing and local immune activation

The urinary bladder is not merely the site of intravesical BCG administration but a specialized mucosal immune niche that may support the initiation of trained-immunity-associated responses. Unlike the respiratory or gastrointestinal mucosa, the healthy bladder is exposed to a relatively low microbial burden while simultaneously facing continuous chemical, osmotic and mechanical stress from urine storage and voiding. Consequently, the bladder has evolved a highly responsive innate immune system capable of rapidly recognizing microbial invasion while maintaining immune tolerance to non-pathogenic urinary components. Rather than serving solely as a passive physical barrier, the urothelium functions as an active immunological interface that senses pathogens, orchestrates leukocyte recruitment, and initiates local immune training (1, 5). Unlike other mucosal tissues, the bladder is uniquely exposed to repeated, controlled therapeutic microbial stimulation, making it a unique clinical setting for investigating tissue-specific immune programming associated with trained immunity. The major components of this local immune-training niche are illustrated in **Figure 3**.

**Figure 3:**
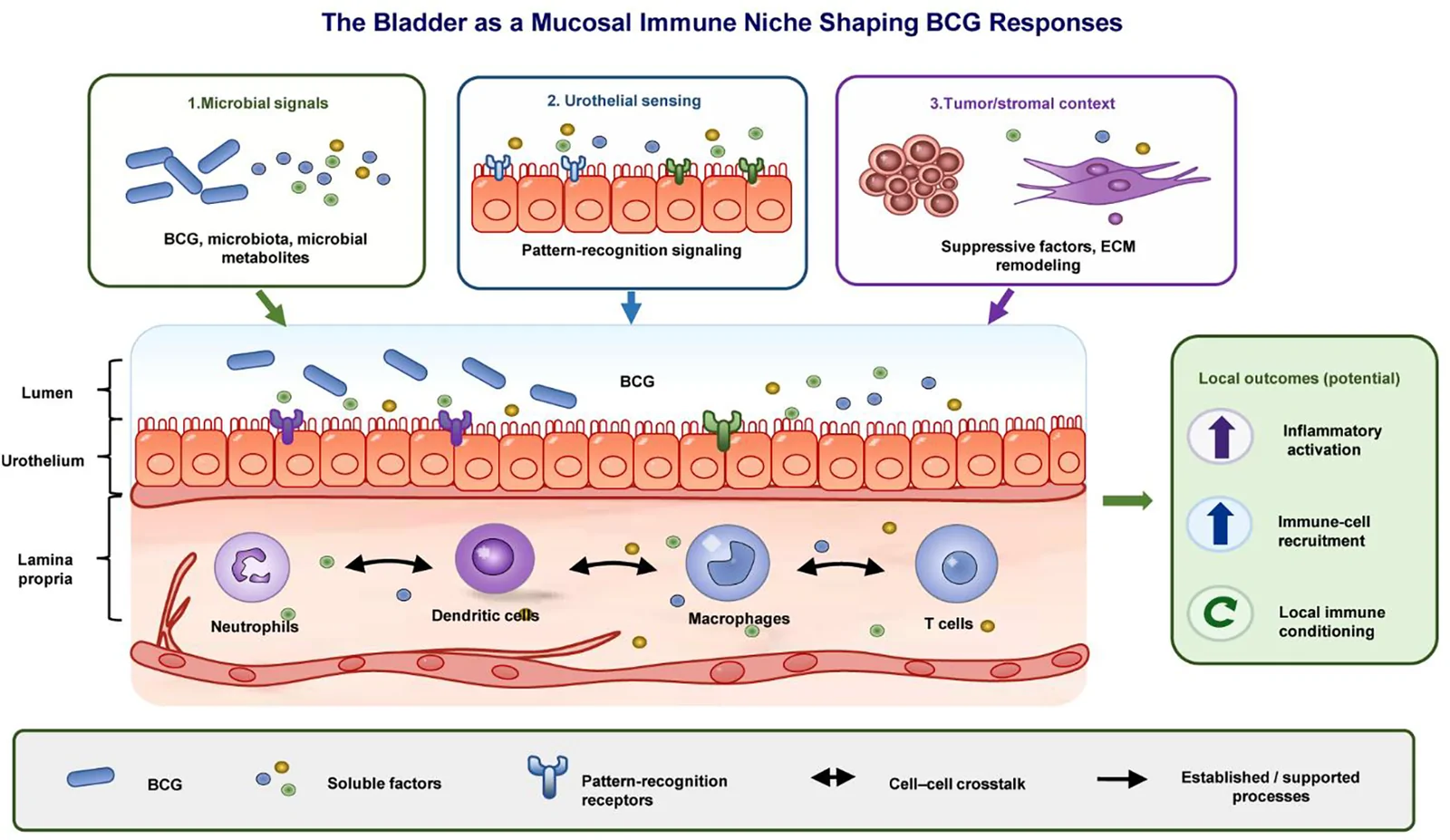
The bladder as a local immune niche shaping BCG responses. The bladder represents a complex mucosal immune environment in which BCG–urothelial interactions are modulated by local microbial, stromal, tumor-derived, and metabolic factors. Intravesical BCG activates pattern-recognition pathways in urothelial and immune cells, promoting cytokine and chemokine production and recruitment of innate immune populations. The urinary microbiota, stromal signals, tumor-derived immunoregulatory factors, and metabolic conditions such as hypoxia, lactate accumulation, and altered lipid metabolism may further shape the magnitude and quality of local immune activation (30). Together, these factors may influence whether BCG exposure results in transient inflammation, productive immune programming, or maladaptive immune responses.

Following intravesical instillation, BCG first encounters the urothelial surface, where it is sensed by both urothelial and tumor cells through multiple pattern-recognition pathways, including Toll-like receptors, NOD2, inflammasome signaling, and potentially the cGAS–STING pathway (1, 5, 23). Following microbial recognition, the urothelium actively translates pathogen sensing into local immune activation by rapidly producing cytokines and chemokines that recruit innate immune cells and initiate antigen presentation. Thus, urothelial sensing represents the initiating event that determines whether acute inflammation is translated into productive trained immunity.

Accumulating evidence suggests that the urothelium should be regarded as an active regulator rather than a passive target of BCG immunotherapy. Beyond initiating innate immune activation, urothelial cells shape the local immune landscape by coordinating cytokine and chemokine production, regulating leukocyte trafficking, and influencing antigen presentation (31, 32). Importantly, bladder cancer cells also participate in this process by sensing microbial stimulation and dynamically modifying cytokine secretion, antigen-presentation capacity, and immune-checkpoint expression. Consequently, the urothelial compartment functions not merely as the site of microbial recognition but as the central organizer of the local immune-training environment, determining whether BCG-induced inflammation is converted into productive trained immunity or maladaptive immune reprogramming.

### 5.2 Myeloid remodeling in the bladder microenvironment

The bladder mucosal niche is further shaped by dynamic interactions among recruited myeloid cells. Rather than functioning as independent effector populations, neutrophils, macrophages, and dendritic cells integrate microbial and tumor-derived signals to determine the quality of local immune responses. While BCG-induced inflammation promotes antigen presentation, cytokine production, and subsequent T-cell activation, the tumor microenvironment simultaneously delivers suppressive cues—including lactate, hypoxia, prostaglandins, and transforming growth factor-β—that can redirect infiltrating myeloid cells toward immunoregulatory phenotypes (33, 34). Consequently, the bladder represents a dynamic immune ecosystem in which productive trained immunity continuously competes with tumor-induced immune suppression, ultimately determining the quality of BCG-induced immune training and therapeutic responsiveness.

### 5.3 Metabolic regulation of local immune training

Another defining feature of the bladder mucosal niche is its distinctive metabolic environment. Unlike most tissues, immune cells recruited into the bladder are directly exposed to urine while simultaneously encountering tumor-associated hypoxia, oxidative stress, lactate accumulation, and altered lipid metabolism. Because metabolic remodeling is essential for trained immunity, these local conditions are likely to influence whether BCG-induced inflammation is translated into durable immune training or diverted toward immune tolerance and suppressive myeloid differentiation (10, 22, 25–27). Accordingly, the bladder should be regarded not merely as the site of BCG instillation but as a metabolically specialized tissue that actively determines the quality and persistence of innate immune training, thereby influencing the durability of therapeutic response.

### 5.4 Urinary microbiota as a potential modifier

Local microbial ecology may further modulate BCG responsiveness. Although the urinary tract was traditionally considered sterile, culture-independent sequencing has identified diverse urinary microbial communities associated with bladder cancer, immune-cell infiltration, and response to intravesical therapy (35, 36). Resident microorganisms may influence pattern-recognition signaling, cytokine production, or competition for epithelial attachment, whereas antibiotic exposure has been associated with altered clinical outcomes following BCG therapy (37). Although the underlying mechanisms remain incompletely understood, these observations suggest that the urinary microbiome constitutes an additional component of the bladder training niche.

Collectively, these observations support the concept of the bladder as a specialized mucosal environment in which epithelial sensing, myeloid-cell programming, metabolism, and microbial ecology may influence whether BCG-induced inflammation is accompanied by durable immune reprogramming. Signals generated within this environment could contribute to systemic hematopoietic responses, forming the proposed initiating component of the bone marrow–bladder axis.

## 6 The proposed bone marrow–bladder axis

### 6.1 Evidence for systemic hematopoietic reprogramming

Emerging evidence that intravesical BCG can induce systemic and hematopoietic changes associated with trained immunity has broadened the conventional understanding of bladder cancer immunotherapy. Traditional models emphasize immune activation within the bladder, where microbial recognition initiates inflammatory signaling and recruits innate and adaptive immune cells. However, this local paradigm alone may not fully account for the persistence of systemic immune alterations observed after treatment (7, 9, 14). These findings raise the possibility that BCG-induced immunity extends beyond the bladder and involves systemic hematopoietic responses.

Direct evidence for this systemic component has recently emerged. Using orthotopic bladder cancer models together with samples from patients receiving intravesical BCG, Daman and colleagues demonstrated that bladder-administered BCG can reprogram hematopoietic stem and progenitor cells (HSPCs), altering myelopoiesis and generating neutrophils, monocytes, and dendritic cells with enhanced antitumor functions (9). Importantly, transplantation of HSPCs from BCG-treated mice transferred enhanced antitumor immunity to recipient animals, providing functional evidence that hematopoietic reprogramming can contribute to durable tumor control. These findings provide mechanistic support for considering the bone marrow as a potential contributor to BCG-induced antitumor immunity rather than merely a source of circulating immune cells.

### 6.2 Bladder-to-bone marrow signaling

The molecular mechanisms underlying bladder-to-bone marrow communication remain incompletely defined. Candidate mediators include circulating cytokines, microbial products, damage-associated signals, and potentially BCG itself, although their relative contributions remain uncertain (9, 10, 13). These signals may promote transcriptional, epigenetic, and metabolic remodeling of HSPCs and thereby alter subsequent myeloid output (10, 13, 22). Because HSPCs are long-lived, such reprogramming provides a plausible mechanism for immune effects that persist beyond the lifespan of individual mature myeloid cells.

### 6.3 A dynamic and temporally organized immune circuit

On this basis, we propose the bone marrow–bladder axis as a conceptual framework in which local BCG exposure and systemic hematopoietic responses are functionally linked. In this model, these signals may reinforce hematopoietic reprogramming, generating altered myeloid populations that subsequently enter the circulation and contribute to remodeling of the bladder tumor microenvironment. Repeated BCG instillations could potentially reinforce this hematopoietic program, although whether such reinforcement contributes to the clinical benefit of maintenance BCG remains unknown. The axis should therefore be viewed as a dynamic and potentially bidirectional immune circuit rather than a strictly local or linear response.Importantly, the proposed axis is unlikely to operate uniformly across patients. Aging, chronic inflammation, metabolic disorders, and tumor-derived suppressive signals may impair HSPC reprogramming or redirect hematopoiesis toward suppressive rather than antitumor myeloid states (10, 22). Thus, variability in BCG response may reflect not only differences in local immune activation but also differences in the establishment, maintenance, or functional direction of hematopoietic training. This possibility provides one biological foundation for the Failure-to-Train framework developed below.

The proposed bone marrow–bladder axis is likely to evolve through temporally overlapping rather than strictly sequential processes. However, the kinetics of HSPC reprogramming, emergence of trained circulating myeloid populations, and their relationship to local and adaptive immune responses remain incompletely defined in patients with NMIBC. Longitudinal sampling of urine, peripheral blood, and tumor tissue will therefore be required to establish the temporal organization of this proposed axis.

Collectively, available evidence supports a model in which local BCG exposure can induce systemic hematopoietic reprogramming, with bone marrow-derived myeloid populations potentially contributing to subsequent antitumor immunity. Nevertheless, the relative contributions of local, circulating, and bone marrow compartments to clinical BCG efficacy remain unresolved. The bone marrow–bladder axis should therefore be regarded as a hypothesis-generating framework integrating emerging clinical and mechanistic observations rather than as an established mechanism of therapeutic response.

## 7 Innate immune memory as a gateway to adaptive antitumor immunity

Trained innate immunity may influence BCG efficacy not only through altered myeloid function but also by shaping downstream adaptive antitumor responses. BCG promotes the accumulation of CD4^+^ and CD8^+^ T cells within the bladder and induces Th1-polarized immunity characterized by IL-12, IFN-γ, and cytotoxic effector activity (1, 5, 14). Within the proposed framework, the relevant question is therefore whether and how reprogrammed innate cells facilitate the transition from microbial sensing to tumor-reactive T-cell immunity.

### 7.1 Dendritic cells and antigen presentation

Dendritic cells provide an important interface between innate and adaptive immunity through antigen acquisition, maturation, costimulatory signaling, and IL-12 production. Recent experimental evidence indicates that BCG-associated hematopoietic reprogramming can increase dendritic-cell production and tumor infiltration and enhance T-cell-dependent tumor control (9, 38).

### 7.2 Myeloid–lymphoid crosstalk

Monocytes, macrophages, and neutrophils may further support this process through cytokine production, immune-cell recruitment, and microenvironmental remodeling, although tumor-derived suppressive signals can redirect these populations toward immunoregulatory states (33, 34, 39). NK cells may provide additional amplification through cytokine production and direct tumor-cell killing, although their specific contribution to trained-immunity-associated adaptive responses remains less well defined.

### 7.3 Adaptive immunity and checkpoint regulation

Clinical observations support the biological relevance of adaptive immune engagement during BCG therapy. Longitudinal immune profiling of patients with NMIBC demonstrated expansion of central-memory CD4^+^ T cells and increased infiltration of activated CD8^+^ T cells during treatment (40). Higher pretreatment densities of non-regulatory T cells and CD8^+^PD-1^+^ T cells were associated with improved recurrence-free survival, although these observations do not establish that trained innate immunity is required for these adaptive responses.

These innate–adaptive interactions also provide a rationale for combining BCG with immune-checkpoint blockade when tumor-reactive T cells are present but functionally constrained (41). However, checkpoint inhibition would not be expected to correct defects occurring earlier in antigen presentation or myeloid function.

Collectively, available evidence supports functional crosstalk between innate and adaptive immunity during BCG therapy, in which trained-immunity-associated myeloid responses may contribute to antigen presentation, cytokine production, and immune-cell recruitment. However, whether trained innate immunity is required for effective adaptive antitumor responses remains uncertain. This unresolved relationship provides the conceptual rationale for the adaptive-transfer stage of the FTT framework discussed below.

## 8 Failure to train: reframing BCG resistance

### 8.1 Five mechanistic stages of failure-to-train

Conceptually, these five potential failure states can be organized into three broader categories: pre-training failure (failure of engagement), failure of immune training (failure of initiation and central training), and post-training failure (failure of execution and adaptive transfer). Importantly, BCG resistance represents a broader biological phenomenon, whereas BCG-unresponsive disease represents a clinically defined category. The FTT framework addresses only selected immune-training-related mechanisms within this broader spectrum and is not intended to replace established clinical classifications or management decisions.

BCG resistance is conventionally classified according to the timing and pathological pattern of tumor persistence or recurrence. Although these clinical definitions are essential for management, they provide limited insight into the biological processes underlying treatment failure. We therefore propose Failure-to-Train (FTT) as a hypothesis-generating framework that organizes immune-training-related vulnerabilities along a proposed bone marrow–bladder immune-training circuit. The framework comprises five potential stages: failure of engagement, failure of initiation, failure of central training, failure of execution, and failure of adaptive transfer (**Figure 4**). These stages represent conceptual mechanistic vulnerabilities rather than clinically validated categories and do not encompass all mechanisms contributing to BCG resistance.

**Figure 4:**
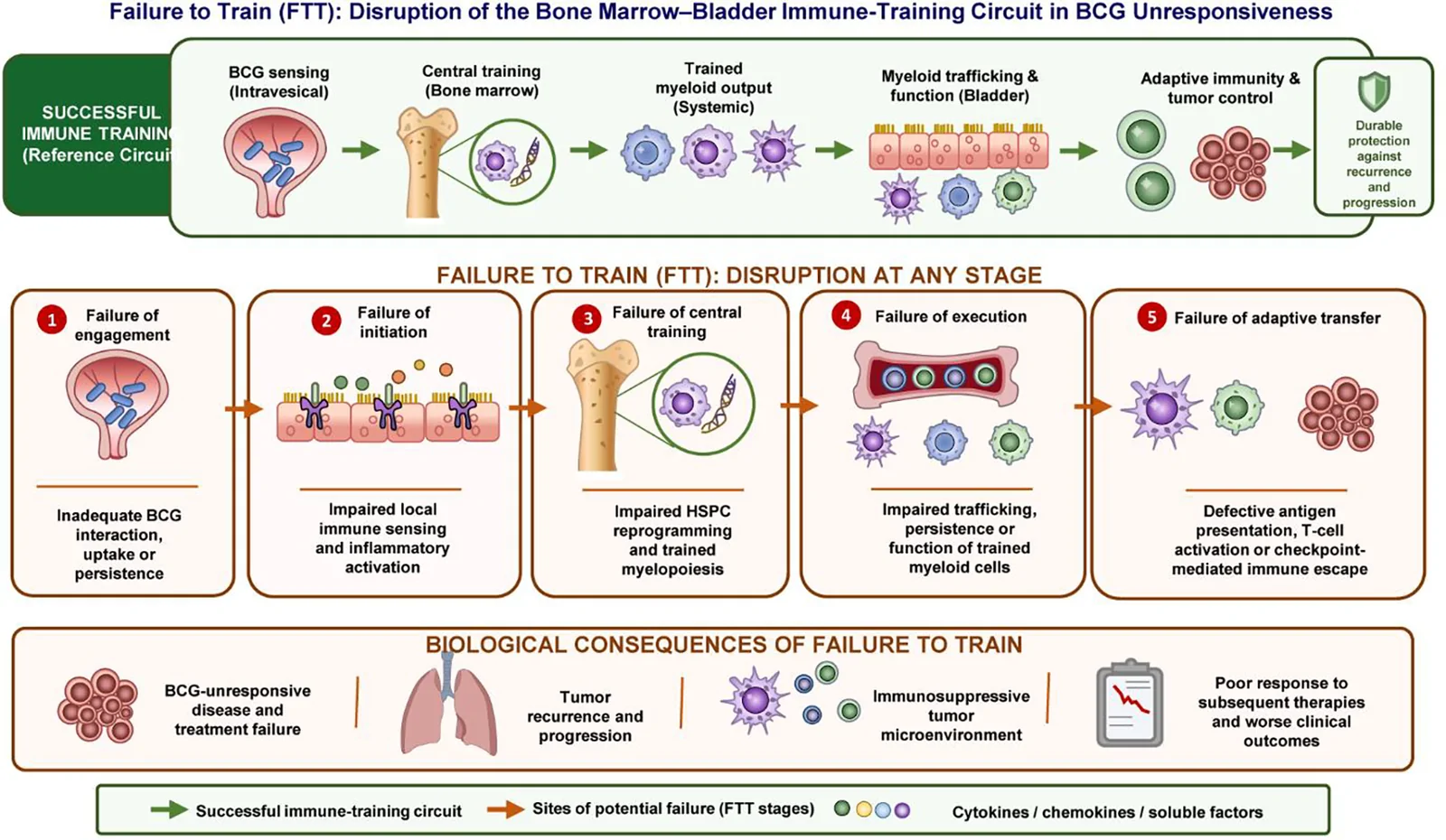
The canonical failure-to-train framework in BCG immunotherapy. The proposed FTT framework comprises five potential failure states: failure of engagement, failure of initiation, failure of central training, failure of execution, and failure of adaptive transfer. These stages represent hypothesis-generating mechanistic vulnerabilities rather than validated biological or clinical subtypes of BCG resistance. Disruption at different stages may lead to ineffective immune training and impaired antitumor responses through distinct biological mechanisms. Conceptually, failure of engagement represents a pre-training vulnerability, failure of initiation and central training involve inadequate establishment of productive immune training, whereas failure of execution and adaptive transfer represent downstream or post-training defects. Detailed candidate mechanisms, measurable indicators, alternative explanations, and potential interventions are summarized in **Table 3**.

Failure of engagement occurs when adequate exposure of urothelial or tumor cells to viable BCG is not achieved. Incomplete BCG exposure, treatment interruption, reduced bacterial viability, impaired urothelial contact, or altered fibronectin-mediated attachment may weaken the initiating stimulus (1, 5, 14). In this setting, the biological defect precedes immune training itself.

Failure of initiation refers to inadequate conversion of BCG engagement into effective local innate activation. Defective pattern-recognition signaling, insufficient inflammatory responses, or development of innate tolerance may prevent acute microbial sensing from generating signals capable of supporting subsequent immune reprogramming (9, 10, 22). Importantly, urinary cytokine release or cystitis-like symptoms alone do not establish that productive training has occurred.

Failure of central training refers to absent, weak, or maladaptive HSPC reprogramming. Aging, chronic inflammation, metabolic disease, previous infections, and tumor-derived systemic signals may alter the bone marrow niche and influence subsequent myelopoiesis (10, 22). Under such conditions, hematopoietic responses may be insufficient or biased toward suppressive rather than antitumor myeloid states. Although experimentally plausible, the contribution of this failure state to clinical BCG resistance remains to be established.

Failure of execution occurs when altered myeloid output is generated but fails to reach the bladder or maintain antitumor function within the tumor microenvironment. Impaired immune-cell recruitment and tumor-derived immunosuppressive signals may limit or redirect the function of monocytes, macrophages, neutrophils, and dendritic cells (42, 43). Thus, myeloid-cell abundance alone cannot be equated with productive immune training.

Failure of adaptive transfer refers to inadequate conversion of innate and myeloid responses into effective tumor-reactive T-cell immunity. Defective dendritic-cell recruitment, antigen presentation, and T-cell infiltration may limit this transition (9), whereas PD-1/PD-L1-mediated inhibition may constrain T-cell activity after priming has occurred (41, 44). Importantly, PD-L1 is a dynamic and context-dependent marker rather than a universal determinant of BCG resistance, and checkpoint-mediated escape represents only one potential mechanism within this stage (41, 44).

### 8.2 Clinical context and boundaries of the FTT framework

The FTT framework should be distinguished from established clinical categories of BCG failure. BCG-unresponsive disease includes BCG-refractory disease and selected early high-grade relapses after adequate BCG exposure, whereas BCG-relapsing disease refers to recurrence after an initial disease-free response (45, 46). These clinically distinct patterns may arise from different combinations of immune-training defects, tumor evolution, microenvironmental remodeling, or adaptive immune escape and should therefore not be assumed to share a single biological mechanism. Importantly, the FTT framework is not intended to replace established clinical definitions of adequate BCG exposure or BCG-unresponsive disease and should not alter guideline-directed management decisions, including timely radical cystectomy when clinically indicated.

Host, tumor, and microbial factors may further modify multiple stages of the proposed FTT framework (10, 35–37). However, associations involving factors such as antibiotic exposure or urinary microbial profiles remain largely observational and should be regarded as candidate modifiers rather than validated determinants of BCG resistance. Importantly, the FTT framework is not mutually exclusive with established mechanisms of BCG resistance. Tumor evolution, altered antigen presentation, checkpoint upregulation, and remodeling of the tumor microenvironment may intersect with individual FTT stages or contribute to treatment failure independently of trained immunity (41, 47, 48). FTT should therefore be viewed as a complementary, hypothesis-generating framework for organizing selected immune-training-related mechanisms rather than as a comprehensive explanation of BCG resistance. These mechanisms may coexist and evolve during treatment. Potential biological defects, candidate biomarkers, and mechanism-guided interventions associated with each FTT stage are summarized in **Table 3**.

**Table 3:** Hypothesis-generating failure-to-train framework for conceptualizing immune-training-related mechanisms of BCG failure.

Failure stage	Biological defect	Candidate mechanisms/causes	Candidate measurable indicators	Alternative explanations	Testable predictions	Potential mechanism-guided interventions
1. Failure of engagement (pre-training condition) (1, 5, 14, 33–35)	Insufficient BCG delivery, persistence, attachment, or local exposure required to initiate immune activation.	Short dwell time; rapid washout; low viable dose; impaired fibronectin interaction; biofilm; concomitant antibiotics; urothelial barrier dysfunction.	Urinary BCG recovery; viability assays; urothelial fibronectin and barrier markers; early local inflammatory readouts.	Adequate BCG exposure may still fail because of tumor-intrinsic resistance or downstream immune defects.	Patients with insufficient engagement will show reduced early innate activation despite adequate treatment intent.	Optimization of BCG delivery, strain selection, dose and dwell time; sustained-release approaches; local delivery technologies.
2. Failure of initiation (5, 9, 10, 14, 22, 58, 59)	Inadequate urothelial or tumor-cell sensing and insufficient early inflammatory activation after BCG exposure.	Reduced TLR/NOD2/STING signaling; impaired NF-κB or inflammasome activation; weak IL-1β, TNF, GM-CSF or chemokine responses; dominant IL-10/TGF-β signaling.	Early urinary cytokines and chemokines; PRR expression; phospho-signaling; inflammasome activation; early immune-cell recruitment.	Tumor immune suppression or altered antigenicity may impair response independently of defective initiation.	Early inflammatory signatures after BCG exposure will predict subsequent immune-training-associated responses.	PRR or STING agonists; engineered BCG; cytokine-based approaches; barrier-directed strategies.
3. Failure of central training (9, 10, 13, 22–27)	Insufficient or maladaptive hematopoietic/HSPC reprogramming and impaired establishment of durable trained myeloid output.	Defective epigenetic or metabolic remodeling; aging; chronic inflammation; metabolic disease; altered marrow niche; suppressive systemic tumor signals.	Circulating progenitor profiling; myeloid trajectories; H3K4me3/H3K27ac and chromatin accessibility; metabolic signatures; heterologous restimulation assays; research-based bone marrow multi-omics.	Peripheral immune dysfunction may reflect systemic inflammation, tumor burden, or treatment-related effects rather than failed HSPC training.	Patients with impaired central training will lack persistent trained-immunity-associated signatures despite BCG exposure.	Candidate trained-immunity agonists; metabolic or epigenetic modulation; HSPC-directed strategies; marrow-niche modulation.
4. Failure of execution (9, 31, 32, 41, 42, 45, 46)	Trained or recruited myeloid cells fail to traffic to, persist within, or exert effective antitumor functions in the bladder.	Defective dendritic-cell maturation; suppressive macrophages; dysfunctional neutrophils; deficient chemokine gradients; impaired CCR/S1PR signaling; vascular or stromal barriers.	Myeloid-cell states; DC maturation markers; functional assays; CXCL9/CXCL10; CCR2/CXCR3; spatial localization of immune cells.	Tumor evolution or local immune exclusion may prevent antitumor activity despite intact systemic immune training.	Discordance between systemic trained-immunity signatures and local bladder immune activity will identify execution failure.	Myeloid reprogramming strategies; DC agonists; chemokine modulation; metabolic intervention; stromal/vascular normalization.
5. Failure of adaptive transfer (9, 41, 43, 44, 47, 50, 51, 56, 57, 60, 61)	Failure of innate immune activation and training to generate durable tumor-reactive adaptive immunity.	Impaired antigen presentation; inadequate costimulation; T-cell exclusion or exhaustion; checkpoint activation; Treg/MDSC-mediated suppression.	MHC and costimulatory molecules; mature DC markers; CD8+ T-cell density; TCR expansion; PD-1/PD-L1; Treg/MDSC abundance.	Tumor-intrinsic antigen loss, clonal evolution, or immune escape may occur independently of trained immunity.	Patients with intact innate activation but defective adaptive transfer will show discordant myeloid and lymphoid immune signatures.	Checkpoint blockade; IL-15-based approaches; T-cell agonists; Treg/MDSC targeting; therapeutic cancer vaccines.

The five Failure-to-Train (FTT) stages represent a hypothesis-generating mechanistic framework rather than validated biological or clinical subtypes. Candidate measurable indicators, alternative explanations, and mechanism-guided intervention strategies are proposed for future experimental and prospective clinical validation.

## 9 Engineering next-generation BCG-based immunotherapies

The emerging biology of trained immunity suggests that next-generation BCG-based therapy should move beyond nonspecific amplification of bladder inflammation. A more rational objective is to control the spatial distribution, magnitude, and duration of immune activation while directing it toward productive hematopoietic training, effective antigen presentation, and durable T-cell-mediated tumor control. This goal may be pursued through recombinant BCG strains, cytokine-based amplification, innate immune agonists, immune-checkpoint combinations, rational chemo-immunotherapy, and biomarker-guided treatment schedules.

### 9.1 Recombinant BCG and strain engineering

One strategy is to redesign the microorganism itself. Conventional BCG substrains differ genetically and phenotypically, with potential differences in immunogenicity and inflammatory activity, but were not developed specifically as cancer therapeutics. Recombinant strains may improve cytosolic antigen delivery, enhance innate immune sensing, or reduce pathogenicity while preserving immunogenicity. VPM1002BC contains a modified BCG backbone designed to facilitate phagosomal escape and antigen presentation. In the phase 1/2 SAKK 06/14 trial, intravesical VPM1002BC showed acceptable tolerability and antitumor activity in patients with NMIBC recurring after conventional BCG (49). However, current evidence is limited to early-phase, single-arm studies, and superiority over conventional BCG in inducing trained immunity or improving clinical outcomes remains to be established in randomized trials.

Within this context, an additional consideration is whether conventional BCG substrains differ in their capacity to induce trained immunity. BCG substrains have accumulated genetic and phenotypic differences during decades of laboratory propagation, which may influence their antigenic composition, immunogenicity, and antitumor activity (50, 51). Comparative preclinical studies have demonstrated strain-dependent differences in direct antitumor effects and cytokine induction (50), while Connaught has been reported to induce stronger Th1-biased responses, BCG-specific CD8^+^ T-cell priming, and T-cell recruitment to the bladder than Tice in experimental models (51). Clinical comparisons of Connaught and Tice have also suggested strain-dependent differences in recurrence outcomes, although the relative efficacy of these substrains appears to be influenced by the use of maintenance therapy (51, 52). Importantly, these immunological and clinical differences should not be equated with differential induction of trained immunity. Although intravesical BCG can induce systemic trained-immunity-associated changes, including altered cytokine responsiveness, monocyte transcriptional programs, and epigenetic remodeling (7), direct head-to-head studies comparing canonical trained-immunity signatures between Tice, Connaught, and other clinically used substrains are currently lacking. It therefore remains unknown whether the observed substrain-dependent differences in immunogenicity translate into quantitatively or qualitatively distinct hematopoietic or epigenetic training programs. Comparative studies incorporating HSPC and monocyte profiling, chromatin accessibility and histone modifications, metabolic reprogramming, and secondary-stimulation responses will be required to address this question.

### 9.2 Innate immune agonists and cytokine-based strategies

A second strategy is to preserve BCG as the immune-priming backbone while restoring downstream effector-cell expansion. The clearest clinical example is nogapendekin alfa inbakicept-pmln, an IL-15 superagonist also known as N-803. IL-15 promotes NK-cell and CD8^+^ T-cell expansion and function. In the QUILT-3.032 study, intravesical N-803 combined with BCG produced durable responses in patients with BCG-unresponsive NMIBC (53). The combination subsequently received US regulatory approval for BCG-unresponsive carcinoma *in situ* with or without papillary tumors, with an FDA analysis reporting a complete response in 62% of evaluable patients (54). Although the study was single-arm, these findings support the principle that BCG resistance does not necessarily mean that the BCG platform should be abandoned in some patients, BCG-mediated innate priming may remain therapeutically useful if downstream NK- and T-cell activity can be restored.

Direct activation of innate immune pathways provides another means of reinforcing insufficient BCG signaling. STING agonists are particularly relevant because STING activation induces type I interferons, dendritic-cell maturation, chemokine production, and adaptive immune priming. Intravesical E7766 showed strong antitumor activity in orthotopic murine NMIBC models resistant to BCG and anti-PD-1 treatment (55). Similarly, VB-85247 induced durable tumor regression, protected against tumor rechallenge, and enhanced anti-PD-1 activity in preclinical models (56). These findings support local STING activation as a strategy to overcome defective microbial sensing or dendritic-cell activation. Nevertheless, both agents remain predominantly supported by preclinical evidence, and their optimal dosing, safety profiles, and integration with BCG require clinical evaluation.

Other pattern-recognition pathways, including TLRs and NOD2, may also be pharmacologically manipulated. In principle, defined innate agonists could be combined with BCG to reinforce a defective stage of the immune response. However, stronger inflammation is not necessarily more effective. An intervention that increases urinary cytokines without inducing productive HSPC reprogramming, dendritic-cell function, or tumor-reactive T-cell responses may add toxicity without improving tumor control. Future combinations should therefore be evaluated using functional measures of trained immunity and innate-to-adaptive transfer rather than acute inflammatory readouts alone.

### 9.3 BCG–checkpoint inhibitor combinations

Immune-checkpoint blockade targets a later stage of the antitumor immune response. BCG can recruit and activate T cells but may also induce compensatory inhibitory pathways, including PD-1/PD-L1 signaling, providing a rationale for combining BCG with checkpoint blockade when tumor-reactive T-cell responses are present but constrained (41). Recent phase III trials, however, demonstrate that the clinical benefit of BCG–checkpoint combinations is not uniform across agents and treatment regimens. In the ALBAN (GETUG-AFU 37) trial, the addition of intravenous atezolizumab to BCG in BCG-naïve high-risk NMIBC did not improve event-free survival compared with BCG alone (HR 0.98, 95% CI 0.71–1.36) and was associated with increased treatment-related toxicity (57). In contrast, the phase III CREST trial demonstrated that subcutaneous sasanlimab combined with BCG induction and maintenance significantly improved event-free survival compared with BCG induction and maintenance alone (HR 0.68, 95% CI 0.49–0.94), with estimated 36-month event-free survival rates of 82.1% and 74.8%, respectively (58). Similarly, the phase III POTOMAC trial showed that intravenous durvalumab combined with BCG induction and maintenance significantly improved disease-free survival compared with BCG induction and maintenance alone (HR 0.68, 95% CI 0.50–0.93) (59). Durvalumab plus BCG subsequently received US FDA approval on May 28, 2026, for adults with BCG-naïve high-risk NMIBC (60). These contrasting outcomes argue against a uniform class-wide effect of adding immune-checkpoint blockade to BCG and suggest that differences in checkpoint agent, route and duration of administration, BCG exposure and maintenance schedule, patient population, and other trial-specific factors may influence therapeutic outcomes. Importantly, these trials did not directly assess trained-immunity endpoints or demonstrate correction of a specific Failure-to-Train stage; therefore, their clinical efficacy should not be interpreted as mechanistic validation of the proposed FTT framework. Within the proposed framework, BCG may provide immune priming and trained-immunity-associated signals, whereas ICIs primarily act downstream by releasing inhibitory constraints on tumor-reactive T cells (9, 41). This distinction provides a biological rationale for a sequential strategy in which initial BCG exposure supports a model local innate activation, trained myelopoiesis, antigen presentation, and T-cell priming before checkpoint blockade is introduced. Although concurrent administration may maximize temporal overlap between BCG-induced immune priming and checkpoint blockade, available clinical evidence has not established that this approach is superior to sequential administration, and concurrent combination therapy may increase treatment-related toxicity (57). Conversely, from a mechanistic perspective, delaying ICI therapy until overt clinical BCG failure could also be suboptimal if checkpoint-mediated adaptive resistance emerges during treatment (41, 44). These observations raise the hypothesis that biomarker-guided sequencing may warrant prospective evaluation, in which checkpoint blockade is introduced after evidence of productive immune priming but before irreversible immune escape or tumor progression. Longitudinal assessment of T-cell activation and recruitment, tumor-infiltrating CD8^+^ T cells, and dynamic PD-1/PD-L1 expression may help define this therapeutic window (40, 41, 44). Importantly, the optimal timing of BCG–ICI combinations has not yet been established clinically, and prospective trials directly comparing concurrent, sequential, and biomarker-triggered strategies are required.

### 9.4 Local combination and alternative therapeutic strategies

Combining BCG with local chemotherapy or tumor-disrupting agents offers another opportunity. Cytotoxic treatment may reduce tumor burden, release tumor antigens, and remodel the immune microenvironment, whereas BCG provides inflammatory and antigen-presenting signals. The 2026 ANZUP 1301 randomized trial compared BCG plus mitomycin with BCG alone in BCG-naïve NMIBC (61). In the overall study population, the combination did not demonstrate clear superiority in disease-free survival (HR 0.87, 95% CI 0.65–1.16), although the regimen may have potential BCG-sparing implications. These findings caution against assuming that adding local chemotherapy uniformly enhances BCG efficacy and highlight the importance of treatment schedule, BCG exposure, and patient selection. Importantly, trained-immunity endpoints were not directly assessed in ANZUP 1301, and its clinical findings therefore should not be interpreted as evidence that a specific FTT stage was modified.

Gene-based and oncolytic viral therapies also provide conceptual lessons for BCG development, even when they are not themselves BCG products. These approaches can generate local interferon signaling, tumor-cell lysis, and antigen release while avoiding live mycobacterial exposure. Their emergence demonstrates that the major functions historically bundled within BCG—microbial sensing, local inflammation, antigen release, and adaptive priming—can be separated and potentially recombined according to the dominant biological defect in each patient.

### 9.5 Toward mechanism-guided combination therapy

The proposed bone marrow–bladder axis also suggests that BCG dosing and maintenance schedules should be reconsidered. Conventional schedules were established empirically, whereas repeated instillations may function as hematopoietic training or booster events. Excessively frequent exposure could promote tolerance or toxicity, while insufficient exposure may fail to stabilize central trained immunity. Future schedules should therefore account for the kinetics of local inflammation, peripheral monocyte reprogramming, HSPC activation, and adaptive immune expansion. Dynamic biomarkers may help determine whether a patient requires further BCG, an innate agonist, cytokine amplification, or checkpoint release rather than uniform continuation of maintenance therapy.

From the perspective of the proposed framework, next-generation BCG-based immunotherapies could be investigated as approaches to modulate distinct components of immune activation, hematopoietic reprogramming, and adaptive antitumor immunity. Four principles are central: improving the quality and safety of microbial sensing; establishing productive rather than maladaptive hematopoietic training; ensuring efficient transfer from trained myeloid cells to tumor-reactive lymphocytes; and selecting interventions according to the stage at which the immune relay is defective. Recombinant BCG, IL-15 amplification, intravesical STING agonists, checkpoint blockade, and rational chemo-immunotherapy each target different components of this circuit. Whether their clinical activity can be improved by matching treatment choice or timing to specific immune defects remains an important hypothesis for prospective testing. Representative next-generation BCG-based therapeutic strategies are summarized in **Table 4**. Selected clinical and translational studies of next-generation BCG-based therapies are summarized in **Supplementary Table S1**.

**Table 4:** Mechanism-guided next-generation strategies for precision BCG-based immunotherapy.

Strategy	Representative approach	Biological rationale	FTT stage primarily targeted	Current evidence and development status	Major limitations
Recombinant BCG (52–55)	VPM1002BC	Modified bacterial biology designed to enhance antigen release, immunogenicity and safety.	Failure of engagement/initiation and antigen presentation	Early clinical evidence; superiority over conventional BCG has not been established in randomized comparisons.	Limited proof of improved efficacy compared with standard BCG; long-term comparative data remain needed.
IL-15 pathway amplification (56, 57)	Nogapendekin alfa inbakicept-pmln (N-803) plus BCG	Expands and activates NK and CD8+ T cells and may strengthen innate-to-adaptive immune transfer after effective immune priming.	Cytokine insufficiency and failure of adaptive transfer	Clinical evidence established for a defined BCG-unresponsive CIS population; broader applications remain under evaluation.	Single-arm pivotal evidence in specific populations; optimal sequencing with BCG and other immunotherapies remains uncertain.
Immune-checkpoint blockade (44, 60, 61)	Pembrolizumab plus BCG and other PD-1/PD-L1 combinations	Restores activity of BCG-primed but checkpoint-restrained T cells.	Failure of adaptive immune transfer and checkpoint-mediated escape	Combination approaches are under clinical evaluation; efficacy appears dependent on effective immune priming and tumor immune context.	Unlikely to overcome absent innate training or severe immune exclusion; systemic immune-related toxicity remains a concern.
STING agonists (58, 59)	Intravesical cyclic dinucleotide or non-nucleotide agonists	Enhance type I interferon signaling, dendritic-cell maturation and T-cell priming.	Failure of sensing, initiation and antigen presentation	Mainly preclinical or early clinical development.	Dose-dependent inflammation, urothelial toxicity and optimal combination strategies require further study.
Metabolic training strategies (22, 25–27)	Modulation of glycolysis, mevalonate pathway or glutaminolysis	May reinforce epigenetic and metabolic programs associated with trained immunity.	Failure of central immune reprogramming	Preclinical and conceptual evidence.	Potential systemic metabolic effects and risk of maladaptive inflammation.
Myeloid- or HSPC-directed therapy (9, 10, 13, 22)	Cytokines, niche modifiers or *in vivo* reprogramming approaches	Promote productive trained myelopoiesis and restoration of myeloid immune function.	Failure of central training	Early preclinical concept.	Potential off-target hematopoietic effects and systemic inflammatory consequences.
Biomarker-guided treatment (7–10, 22, 43, 44)	Integrated urine, peripheral blood and tumor tissue profiling	Identifies dominant immune failure states and may guide mechanism-matched treatment selection.	All FTT stages	Translational research; prospective validation studies are required.	Lack of standardized assays, longitudinal sampling strategies and clinically validated predictive biomarkers.

Strategies are organized according to the proposed Failure-to-Train (FTT) framework. Current evidence and development status reflect the level of clinical or preclinical support, whereas proposed mechanisms and targeted failure stages remain hypothesis-generating and require further validation.

## 10 Biomarkers of productive and maladaptive innate immune training

The clinical management of non-muscle-invasive bladder cancer currently relies primarily on clinicopathological characteristics, including tumor stage, grade, carcinoma *in situ*, recurrence history and previous BCG exposure. Although these parameters are useful for risk stratification, they provide limited information regarding the biological processes that determine treatment success or failure. Similarly, individual biomarkers such as PD-L1 expression, tumor mutational burden or urinary cytokines have shown variable predictive performance across studies. A fundamental limitation of these approaches is that they evaluate isolated features of the immune response rather than the establishment of durable innate immune memory. We propose that biomarker development should move beyond predicting clinical recurrence and instead determine whether immune training has been successfully established, fails to develop, or is diverted toward maladaptive reprogramming following BCG therapy.

Within the FTT framework, biomarkers should be interpreted according to the stage of the immune-training circuit they reflect rather than as isolated predictors of therapeutic outcome. Early events occurring within the bladder can be monitored through urinary inflammatory mediators and immune-cell recruitment, reflecting successful microbial engagement and local innate activation. However, these markers primarily indicate acute inflammation and do not necessarily predict long-term therapeutic benefit. More informative biomarkers are likely to emerge from peripheral immune cells, where ex vivo stimulation assays have demonstrated sustained increases in TNF and IL-1β production following intravesical BCG, consistent with systemic trained immunity (7). Likewise, transcriptional, epigenetic and metabolic signatures of circulating monocytes may provide functional evidence that innate immune reprogramming has occurred rather than merely documenting transient cytokine release (8, 10, 22).

If long-term trained immunity in NMIBC is substantially supported by hematopoietic reprogramming, biomarkers reflecting central hematopoietic responses may be particularly informative. Direct bone marrow sampling is unlikely to be practical in patients with NMIBC, but circulating monocyte and neutrophil phenotypes, dendritic-cell differentiation and transcriptional programs associated with emergency myelopoiesis may serve as surrogate indicators of central immune training. The recent demonstration that intravesical BCG reprograms hematopoietic stem and progenitor cells and continuously generates functionally enhanced myeloid cells provides a strong biological rationale for developing such biomarkers (9). Importantly, increased myeloid-cell numbers alone should not be interpreted as evidence of productive training, because similar changes may also result from tumor-induced suppressive hematopoiesis. Accordingly, future biomarkers should prioritize functional evidence of trained myelopoiesis rather than simple quantification of circulating myeloid populations.

Ultimately, productive trained immunity must be translated into adaptive antitumor immunity within the bladder tumor microenvironment. Accordingly, tissue biomarkers should evaluate not only immune-cell abundance but also immune-cell function and spatial organization. Higher densities of activated CD8^+^ T cells, mature dendritic cells and inflammatory macrophages, together with efficient antigen presentation and coordinated myeloid–lymphoid interactions, are more likely to reflect successful immune training than individual checkpoint molecules alone (40, 41). Conversely, enrichment of myeloid-derived suppressor cells, regulatory macrophages, T-cell exclusion or persistent checkpoint activation may indicate maladaptive training, in which inflammation is sustained but effective tumor immunity fails to develop. Accordingly, tissue biomarkers should be interpreted as indicators of successful innate-to-adaptive immune transfer rather than isolated measurements of immune-cell abundance.

Based on the FTT framework proposed in this Review, we suggest that future biomarkers should distinguish three biologically distinct states of immune training rather than simply classify patients as BCG responders or nonresponders. Insufficient training reflects failure of engagement or initiation, in which inadequate microbial recognition or weak innate activation fails to establish durable immune reprogramming. Representative biomarkers include limited urothelial activation, transient cytokine production and the absence of sustained trained-immunity signatures. Within the proposed framework, a putative productive-training state would be characterized by evidence of hematopoietic reprogramming, functionally competent myeloid responses, effective antigen presentation, and coordinated adaptive immune activation. However, this state has not yet been prospectively validated as a discrete biological or clinical category in NMIBC. Representative biomarkers include evidence of trained myelopoiesis, coordinated myeloid–lymphoid interactions and robust adaptive immune activation. In contrast, maladaptive training reflects failure of execution or adaptive transfer. Although innate activation and inflammatory signaling are preserved, immune training is diverted toward suppressive myeloid differentiation, defective antigen presentation, T-cell exclusion or checkpoint-mediated immune escape, leading to persistent inflammation without effective antitumor immunity. Representative biomarkers include MDSC enrichment, suppressive macrophage polarization, persistent checkpoint activation and impaired antigen presentation. This biologically based classification links biomarker interpretation directly to the stage of immune-training failure, thereby providing a mechanistic framework for precision immunotherapy rather than a purely prognostic classification of treatment outcome. These three states correspond to distinct biological outcomes of the immune-training circuit and may require fundamentally different therapeutic interventions.

Future biomarker strategies should therefore integrate multiple levels of the immune response rather than relying on a single molecular marker. Longitudinal assessment combining urinary immune activation, peripheral innate immune reprogramming and tissue immune remodeling is likely to provide a more accurate representation of the immune-training process than any individual assay. Mechanism-guided biomarker stratification may facilitate precision immunotherapy by matching therapeutic interventions to the dominant stage of FTT rather than to conventional clinical response categories. Conversely, patients who successfully establish trained immunity but subsequently develop adaptive immune suppression may be more suitable candidates for immune-checkpoint blockade.

Overall, biomarkers of BCG immunotherapy should evolve from static predictors of recurrence toward dynamic indicators of immune memory. Rather than simply asking whether inflammation has occurred, future studies should determine whether biomarkers can identify putative defects at different stages of the proposed immune-training circuit. Such a multidimensional strategy may ultimately enable mechanism-guided selection of BCG maintenance, trained-immunity agonists, myeloid-targeted therapies and immune-checkpoint blockade according to the dominant biological defect underlying FTT. Candidate measurable indicators and representative detection approaches associated with different FTT stages are summarized in **Table 3**.

## 11 Experimental models and methodological priorities

Experimental validation of the proposed bone marrow–bladder axis and FTT framework will require models capable of resolving the temporal, spatial, and functional changes associated with BCG treatment. Although recent studies provide mechanistic evidence for BCG-associated hematopoietic reprogramming, standardized experimental systems and validated endpoints for assessing immune training remain limited.Orthotopic immunocompetent bladder cancer models remain particularly important because they preserve intravesical BCG delivery and relevant tumor–immune interactions. Recent studies using such models have enabled mechanistic investigation of hematopoietic reprogramming following bladder-administered BCG (9). However, differences in tumor genetics, BCG strains, treatment schedules, and host immune backgrounds complicate comparisons across studies and limit direct extrapolation to human NMIBC. Standardized models that incorporate clinically relevant induction and maintenance schedules will therefore be important for reproducible mechanistic studies.

Longitudinal sampling will be essential because static endpoint analyses provide only snapshots of a dynamic immune response. Serial collection of urine, peripheral blood, and tumor tissue before treatment and at defined points during induction and maintenance therapy could clarify the kinetics and persistence of BCG-associated immune reprogramming. Where experimentally feasible, paired bone marrow analyses could provide more direct assessment of HSPC changes, although routine bone marrow sampling is unlikely to be practical in clinical studies.Single-cell and spatial approaches may help distinguish changes in immune-cell abundance from changes in functional state. Single-cell transcriptomic, epigenomic, and metabolic profiling could characterize HSPC and myeloid-cell states associated with BCG exposure, whereas spatial analyses of bladder tumors could determine whether these states are accompanied by appropriate localization of antigen-presenting cells and lymphocytes within the tumor microenvironment. Such approaches may also help distinguish productive immune reprogramming from tolerance or suppressive myeloid states.Functional validation will be equally important because phenotypic or molecular signatures alone cannot establish trained immunity. Ex vivo restimulation assays can assess altered responsiveness of circulating monocytes, while lineage-tracing, adoptive-transfer, depletion, or transplantation approaches in experimental models can test the functional contribution of specific hematopoietic and myeloid populations. Importantly, candidate trained-immunity signatures should be linked to measurable antitumor activity rather than interpreted solely from cytokine production or cell abundance.

Prospective human validation remains the critical next step. Current clinical evidence for BCG-associated systemic immune training is limited by small cohorts, heterogeneous sampling schedules, and the absence of validated assays that directly demonstrate HSPC reprogramming or completion of the proposed immune-training pathway in patients with NMIBC. Future studies should integrate longitudinal urine, blood, and tumor analyses with clinical outcomes to determine whether candidate immune-training states are reproducible, biologically distinct, and associated with BCG response or resistance. Until such validation is available, these states should be regarded as investigational rather than clinically actionable biomarkers. Together, these approaches will be required to determine whether the proposed FTT states represent biologically distinct and clinically relevant mechanisms of BCG failure.

## 12 Safety, biological trade-offs and clinical translation

Intravesical BCG is generally well tolerated, but local urinary symptoms and transient systemic reactions are common, whereas severe complications are uncommon. Importantly, treatment-related inflammation or toxicity should not be interpreted as evidence of productive trained immunity. Robust inflammatory responses may occur without durable antitumor benefit, emphasizing the need to distinguish nonspecific immune activation from functionally effective immune reprogramming.

Therapeutic manipulation of trained immunity is likely to require a carefully defined biological window. Insufficient reprogramming may fail to support effective antitumor immunity, whereas excessive or persistent innate activation could contribute to chronic inflammation or immune tolerance (10, 12) Thus, the therapeutic objective should be appropriately regulated immune reprogramming rather than maximal inflammatory activation. This consideration may be particularly relevant to next-generation BCG formulations and innate immune agonists, for which greater immunogenicity may not necessarily translate into greater therapeutic benefit.

The therapeutic window may also vary among patients. Age, metabolic dysfunction, chronic inflammation, prior microbial exposures, and other host factors may influence baseline innate immune responsiveness and the magnitude or duration of BCG-associated immune reprogramming (10). However, the clinical relevance of these factors for selecting treatment intensity or duration remains unvalidated.

A potential safety concern is that hematopoietic reprogramming may extend immune activation beyond the bladder. Because trained immunity can enhance responses to heterologous stimuli, excessive or persistent activation could theoretically promote systemic inflammation or off-target tissue injury (10, 12). However, direct evidence that BCG-associated hematopoietic training causes clinically significant systemic immune pathology in patients with NMIBC remains limited. This possibility should therefore be regarded as a safety consideration requiring prospective evaluation rather than an established clinical consequence. Future clinical trials should integrate longitudinal immune and safety monitoring with conventional oncological endpoints. Standardized analyses of urine, peripheral blood, and tumor tissue may help determine whether therapeutic benefit is associated with durable immune reprogramming while simultaneously identifying excessive or maladaptive inflammatory responses. Such studies will be necessary to define a clinically meaningful therapeutic window for trained-immunity-directed interventions. Successful clinical translation will therefore depend on balancing antitumor immune reprogramming against inflammatory toxicity rather than maximizing immune activation.

## 13 Future directions for validating the failure-to-train framework

A major priority is to establish the causal contribution, durability, and kinetics of hematopoietic reprogramming during intravesical BCG therapy. Although recent experimental evidence supports BCG-associated HSPC reprogramming and altered myelopoiesis (9), its necessity for therapeutic efficacy in patients with NMIBC has not been established. Prospective longitudinal studies should determine the timing and persistence of these changes and clarify whether induction and maintenance BCG produce similar, cumulative, or qualitatively distinct immune effects.

A second priority is to define experimentally what constitutes productive, insufficient, or maladaptive immune training. Functional, epigenetic, metabolic, and transcriptional criteria should be integrated rather than relying on cytokine production or immune-cell abundance alone. Studies should also determine the relative contributions of specific HSPC, myeloid, and tissue-resident populations to antitumor activity and whether suppressive states represent failed training, competing tumor-driven programming, or biologically distinct processes.

Larger multicenter studies should determine how host, tumor, microbiome, BCG strain, dose, and treatment schedule influence immune-training-associated responses and clinical outcomes. Such studies should establish whether candidate immune states are reproducible across patient populations, precede clinical recurrence, and provide information beyond conventional clinicopathological predictors.

Ultimately, the decisive test of the FTT framework will be whether biologically distinct failure states can be prospectively identified and whether this information improves treatment selection or clinical outcomes. Mechanism-guided intervention studies should therefore test whether correction of a candidate defect restores antitumor immunity without unacceptable toxicity. Until such evidence is available, FTT should remain a hypothesis-generating framework rather than a validated biological or clinical classification.

## 14 Conclusions and perspectives

Intravesical BCG remains the cornerstone of immunotherapy for non-muscle-invasive bladder cancer, yet its clinical efficacy cannot be fully explained by transient local inflammation alone. Direct observations in patients with NMIBC and mechanistic evidence from experimental models suggest that intravesical BCG can be accompanied by persistent systemic innate immune alterations, including changes in circulating myeloid responses and hematopoietic programming (7, 9, 14). These findings raise the possibility that systemic trained-immunity-associated processes may contribute to BCG-induced antitumor immunity alongside established local mechanisms. However, their causal contribution to clinical efficacy and their relationship with adaptive antitumor immunity remain incompletely defined.

In this Review, we propose the bone marrow–bladder axis as a hypothesis-generating conceptual framework linking local microbial sensing with systemic hematopoietic responses that may contribute to downstream myeloid and adaptive antitumor immunity. Building on this framework, we introduce Failure-to-Train as a conceptual model for organizing selected immune-training-related mechanisms that may contribute to BCG resistance. The proposed framework comprises five potential failure states—failure of engagement, failure of initiation, failure of central training, failure of execution, and failure of adaptive transfer—which remain mechanistic hypotheses rather than validated biological or clinical subtypes of BCG resistance. If prospectively validated, this framework may also inform biomarker development and mechanism-guided therapeutic strategies. Rather than relying solely on isolated inflammatory markers, future studies could investigate whether integrated measures of local immune activation, systemic innate reprogramming, myeloid function, and adaptive immunity can distinguish biologically meaningful patterns of BCG response or failure. Whether such information can ultimately guide the selection or timing of engineered BCG, innate immune agonists, cytokine-based therapies, or immune-checkpoint inhibitors remains to be determined.Despite the conceptual advances proposed in this Review, several fundamental questions remain unresolved, including how bladder-derived signals reprogram the bone marrow, how long trained immunity persists, which myeloid populations contribute to durable tumor control, and how productive training can be distinguished from immune tolerance or suppressive myelopoiesis. Addressing these questions will require longitudinal studies integrating clinical outcomes with functional immune profiling and multi-omics analyses.

Overall, emerging evidence supports trained immunity as a potentially important component of the complex immune response to intravesical BCG rather than an established central mechanism of therapeutic efficacy. Direct human studies demonstrate persistent systemic innate immune alterations after treatment, whereas mechanistic bladder-cancer models provide stronger causal evidence linking hematopoietic reprogramming with antitumor immunity. Evidence from systemic BCG vaccination and other trained-immunity settings provides additional biological support but should be regarded as extrapolative. On this basis, the proposed bone marrow–bladder axis and FTT framework are proposed as hypothesis-generating models that integrate these observations and generate testable predictions. Prospective human studies and validated longitudinal assays will be required to determine whether the proposed immune-training circuit contributes causally to BCG responsiveness and can ultimately support biomarker-guided therapy.

## Supplementary material

Supplementary Table 1 Selected clinical and translational studies of next-generation BCG-based therapies in NMIBC. This table summarizes clinical and translational studies of next-generation BCG-based therapies. The potential Failure-to-Train (FTT) stage addressed is a mechanistic interpretation based on the proposed framework and does not indicate that a specific trial has prospectively validated correction of an FTT stage. Trained-immunity-related endpoints refer to direct functional, transcriptional, epigenetic, metabolic, or hematopoietic measurements designed to assess trained immunity.

Supplementary Table 2 Literature-search and evidence-selection strategy for this structured narrative review.
