# Supplementary material for: Reprogramming innate immune memory in bladder cancer: the proposed bone marrow–bladder axis and next-generation BCG-based immunotherapies
Source: Front Immunol. 2026 Oct 6;17:1945559. doi: 10.3389/fimmu.2026.1945559 (PMC13681358; doi:10.3389/fimmu.2026.1945559)
Supplement: Supplementary file 1 [file Table1.doc]

**Supplementary Table S1. Selected clinical and translational studies of next-generation BCG-based therapies in NMIBC**

| Study/trial | Registration | Phase/design | Patient population | Intervention | Comparator and BCG schedule | Primary endpoint | Principal efficacy and safety results | Regulatory status | Potential FTT stage addressed | Trained-immunity-related endpoints |
| --- | --- | --- | --- | --- | --- | --- | --- | --- | --- | --- |
| SAKK 06/14 [50] | NCT02371447 | Phase I/II, multicenter, open-label, single-arm | Recurrent intermediate- or high-risk NMIBC after conventional BCG | Intravesical recombinant VPM1002BC | No comparator; 6 weekly induction instillations with protocol-defined maintenance for 1 year | Bladder recurrence-free rate at 60 weeks | RFR 49.3% at 60 weeks; 47.4% at 2 years and 43.7% at 3 years; grade ≥3 treatment-related AEs 4.8%; no grade ≥4 treatment-related AEs | Not approved for NMIBC | Failure of engagement/initiation and antigen presentation | Not specifically assessed |
| QUILT-3.032 [54,55] | NCT03022825 | Phase II/III, multicohort, open-label, single-arm | BCG-unresponsive high-grade NMIBC; pivotal CIS cohort | Intravesical N-803 (nogapendekin alfa inbakicept-pmln) plus BCG | No comparator; 6-week induction, optional second induction, and maintenance courses | Complete response and duration of response in CIS cohort | FDA efficacy population CR 62%; 58% of responders maintained response ≥12 months and 40% ≥24 months; mainly urinary adverse events | FDA approved with BCG for BCG-unresponsive NMIBC with CIS ± papillary tumors | Cytokine insufficiency and adaptive transfer | Not specifically assessed |
| KEYNOTE-676 | NCT03711032 | Phase III, randomized, comparator-controlled, multicohort | High-risk NMIBC persistent/recurrent after BCG induction or BCG-naive | Pembrolizumab plus BCG | BCG-based comparator according to cohort; protocol-defined BCG induction/maintenance | Cohort-specific efficacy endpoints including CR/EFS | Active/not recruiting; no study results posted as of August 2026 | No approval for pembrolizumab plus BCG in this setting | Adaptive immune escape/checkpoint-mediated suppression | Not specifically assessed |
| ALBAN/GETUG-AFU 37 [58] | NCT03799835 | Phase III, randomized, open-label, international | BCG-naive high-risk NMIBC | Atezolizumab plus BCG | BCG alone; protocol-defined induction and maintenance | Investigator-assessed EFS | No EFS benefit: HR 0.98 (95% CI 0.71–1.36; P=0.9106); grade ≥3 treatment-related AEs 22.7% vs 8.8% | Not approved for this indication | Adaptive immune escape/checkpoint modulation | Not specifically assessed |
| CREST [59] | NCT04165317 | Phase III, randomized, open-label, three-arm global study | BCG-naive high-risk NMIBC | Sasanlimab plus BCG | BCG induction and maintenance comparator | Investigator-assessed EFS | EFS improved: HR 0.68 (95% CI 0.49–0.94); grade ≥3 treatment-related AEs 29.1% vs 6.3% | No FDA approval for this NMIBC combination as of August 2026 | Adaptive immune escape/checkpoint modulation | Not specifically assessed |
| POTOMAC [60,61] | NCT03528694 | Phase III, randomized, open-label, three-arm global study | BCG-naive high-risk NMIBC after TURBT | Durvalumab plus BCG | BCG induction and maintenance comparator | Investigator-assessed DFS | DFS improved: HR 0.68 (95% CI 0.50–0.93; P=0.015); grade 3–4 treatment-related AEs 21% vs 4% | FDA approved May 28, 2026 for adults with BCG-naive high-risk NMIBC | Adaptive immune escape/checkpoint modulation | Not specifically assessed |
| ANZUP 1301 [62] | ACTRN12613000513718 | Phase III, randomized, open-label | BCG-naive NMIBC (high-grade pTa or any-grade pT1; concurrent CIS allowed) | Intravesical BCG plus mitomycin | BCG alone; sequential BCG-mitomycin versus standard BCG schedule | DFS | No clear superiority: 2-year DFS 75% vs 71%; HR 0.87 (95% CI 0.65–1.16; P=0.3); grade 3–5 AEs occurred in 43 vs 37 patients | No distinct regulatory approval for the combination | Potential modulation of immune priming and treatment exposure rather than a defined FTT correction | Not specifically assessed |

This table summarizes clinical and translational studies of next-generation BCG-based therapies. The potential Failure-to-Train (FTT) stage addressed is a mechanistic interpretation based on the proposed framework and does not indicate that a specific trial has prospectively validated correction of an FTT stage. Trained-immunity-related endpoints refer to direct functional, transcriptional, epigenetic, metabolic, or hematopoietic measurements designed to assess trained immunity.

**Table S2. Literature-search and evidence-selection strategy for this structured narrative review.**

| **Item** | **Description** |
| --- | --- |
| Review type | Structured narrative review |
| Databases searched | PubMed/MEDLINE, Web of Science, and Scopus |
| Search period | From database inception to August 15, 2026 |
| Final search date | August 15, 2026 |
| Language restriction | English |
| Core disease terms | “bladder cancer”; “non-muscle-invasive bladder cancer”; “NMIBC” |
| BCG-related terms | “Bacillus Calmette-Guérin”; “BCG”; “BCG resistance”; “BCG-unresponsive” |
| Trained-immunity terms | “trained immunity”; “innate immune memory”; “hematopoietic stem and progenitor cells”; “HSPC”; “myelopoiesis”; “epigenetic reprogramming”; “metabolic reprogramming” |
| Tumor-immunity and therapeutic terms | “tumor microenvironment”; “immune checkpoint” |
| Search approach | Search terms were used individually and in relevant combinations. Reference lists of relevant articles were additionally screened to identify potentially eligible studies. |
| Eligible evidence | English-language original research articles, clinical trials, translational studies, and mechanistic studies relevant to BCG-induced immunity, trained immunity, bladder cancer immunobiology, BCG resistance, biomarkers, and emerging BCG-based therapeutic strategies |
| Animal and in vitro studies | Included when they provided mechanistic evidence relevant to trained immunity, HSPC reprogramming, the proposed bone marrow–bladder axis, or the FTT framework |
| Clinical studies | Prioritized for therapeutic efficacy, safety, biomarkers, treatment outcomes, and clinically relevant immune responses |
| Narrative reviews | Used primarily to provide background context and identify relevant primary literature |
| Conference abstracts | Excluded |
| Preprints | Excluded |
| Other exclusion criteria | Non-English-language publications and studies unrelated to the central themes of the review |
| Study selection | Studies were selected according to relevance to the major themes of the review, with consideration of mechanistic rigor, clinical relevance, methodological quality, and consistency with independent evidence |
| Evidence prioritization | Particular attention was given to studies directly examining BCG-induced trained immunity, systemic innate immune reprogramming, HSPC responses, myeloid-cell remodeling, and innate-to-adaptive immune interactions |
| Quantitative synthesis | Not performed |
| Formal systematic risk-of-bias assessment | Not performed because this article was designed as a structured narrative review rather than a systematic review |
